# A Case-Control of Patients with COVID-19 to Explore the Association of Previous Hospitalisation Use of Medication on the Mortality of COVID-19 Disease: A Propensity Score Matching Analysis

**DOI:** 10.3390/ph15010078

**Published:** 2022-01-08

**Authors:** Jaime Monserrat Villatoro, Gina Mejía-Abril, Lucía Díaz García, Pablo Zubiaur, María Jiménez González, Guillermo Fernandez Jimenez, Inés Cancio, José Ramón Arribas, Carmen Suarez Fernández, Jesús Mingorance, Julio García Rodríguez, José Ramón Villagrasa Ferrer, Antonio J. Carcas, Jesús Frías, Francisco Abad-Santos, Alberto M. Borobia, Elena Ramírez, on behalf of the COVID@HULP Working Group and Other Collaborators from Hospital Universitario de la Princesa

**Affiliations:** 1 Clinical Pharmacology Department, La Paz University Hospital-IdiPAZ, Universidad Autónoma de Madrid, 28046 Madrid, Spain; jaimemonserratvillatoro@gmail.com (J.M.V.); ldiazg@salud.madrid.org (L.D.G.); antonio.carcas@uam.es (A.J.C.); jesus.frias@uam.es (J.F.); 2 Clinical Pharmacology Department, Hospital Universitario de La Princesa, Instituto Teófilo Hernando, Faculty of Medicine, Universidad Autónoma de Madrid (UAM), Instituto de Investigación Sanitaria La Princesa (IP), 28006 Madrid, Spain; ginapaola.mejia@salud.madrid.org (G.M.-A.); pablo.zubiaur@salud.madrid.org (P.Z.); 3 Clinical Trial Unit, La Paz University Hospital-IdiPAZ, 28046 Madrid, Spain; jimenezglezmaria@gmail.com; 4 Medical Information Unit, Instituto de Investigación Sanitaria La Princesa (IP), Hospital Universitario de La Princesa, 28006 Madrid, Spain; gfjimenez@salud.madrid.org (G.F.J.); Ines.Cancio@dxcfds.com (I.C.); 5 Internal Medicine Department, La Paz University Hospital-IdiPAZ, Universidad Autónoma de Madrid, 28046 Madrid, Spain; joser.arribas@salud.madrid.org; 6 Internal Medicine Department, Hospital Universitario de La Princesa, Universidad Autónoma de Madrid, 28006 Madrid, Spain; csuarezf@salud.madrid.org; 7 Microbiology Department, La Paz University Hospital-IdiPAZ, 28046 Madrid, Spain; jesus.mingorance@idipaz.es (J.M.); jgarciarodriguez@salud.madrid.org (J.G.R.); 8 Preventive Medicine Department, Hospital Universitario de La Princesa, Faculty of Medicine, Instituto de Investigación Sanitaria La Princesa (IP), Universidad Autónoma de Madrid (UAM), 28006 Madrid, Spain; joseramon.villagrasa@salud.madrid.org

**Keywords:** coronavirus disease 2019 (COVID-19), severe acute respiratory syndrome coronavirus 2 (SARS-CoV-2), hospitalisation, mortality, previous medication, risk factor, propensity score matching analysis

## Abstract

Data from several cohorts of coronavirus disease 2019 (COVID-19) suggest that the most common comorbidities for severe COVID-19 disease are the elderly, high blood pressure, and diabetes; however, it is not currently known whether the previous use of certain drugs help or hinder recovery. This study aims to explore the association of previous hospitalisation use of medication on the mortality of COVID-19 disease. A retrospective case-control from two hospitals in Madrid, Spain, included all patients aged 18 years or above hospitalised with a diagnosis of COVID-19. A Propensity Score matching (PSM) analysis was performed. Confounding variables were considered to be age, sex, and the number of comorbidities. Finally, 3712 patients were included. Of these, 687 (18.5%) patients died (cases). The 22,446 medicine trademarks used previous to admission were classified according to the ATC, obtaining 689 final drugs; all of them were included in PSM analysis. Eleven drugs displayed a reduction in mortality: azithromycin, bemiparine, budesonide-formoterol fumarate, cefuroxime, colchicine, enoxaparin, ipratropium bromide, loratadine, mepyramine theophylline acetate, oral rehydration salts, and salbutamol sulphate. Eight final drugs displayed an increase in mortality: acetylsalicylic acid, digoxin, folic acid, mirtazapine, linagliptin, enalapril, atorvastatin, and allopurinol. Medication associated with survival (anticoagulants, antihistamines, azithromycin, bronchodilators, cefuroxime, colchicine, and inhaled corticosteroids) may be candidates for future clinical trials. Drugs associated with mortality show an interaction with the underlying conditions.

## 1. Introduction

Data from several cohorts of coronavirus disease 2019 (COVID-19) suggest that the most common comorbidities for severe COVID-19 disease are the elderly, high blood pressure, and other comorbidities; however, it is not currently known whether certain drugs help or hinder recovery [1,2,3,4,5,6,7]. There is evidence that severe acute respiratory syndrome coronavirus-2 (SARS-CoV-2) binds to the cellular receptor for angiotensin-converting enzyme 2 (ACE2) to inoculate itself. The ACE2 receptor is expressed in a high percentage of lung cells. It is also visible in other extrapulmonary tissues, such as the endothelium, heart, kidney, and intestine, which casts doubt on the use of drugs that may impact the renin-angiotensin-aldosterone axis by increasing the probability of acquiring infection. Paradoxically, blocking the renin-angiotensin pathway can attenuate the damage that depends on the expression of ACE2 [8]. Likewise, the use of thiazolidinediones or nonsteroidal anti-inflammatory drugs (NSAIDs) has been associated between mortality from COVID-19 and ACE-inhibitors (ACEI) or angiotensin receptor blockers (ARB) after adjustment for a range of potential confounders [9,10]. Other studies reached similar conclusions or showed beneficial effects [11,12]. In addition, other medications could act as a protector against infection or its severity. Several reviews have proposed candidates to study in clinical trials [13,14,15]. As newer interventions will take months or years to develop, to detail the pool of existing therapeutic options, the principles behind their use to treat COVID-19, current application, and adverse effects could offer candidates for clinical trials [16]. 

The study aimed to explore the association of previous hospitalisation and use of medication with the mortality of patients hospitalised in the cohort of patients with COVID-19 of La Paz University Hospital and La Princesa University Hospìtal (Madrid, Spain).

## 2. Results

### 2.1. Case-Control Results

#### 2.1.1. Characteristics of the Patients

**Figure 1:**
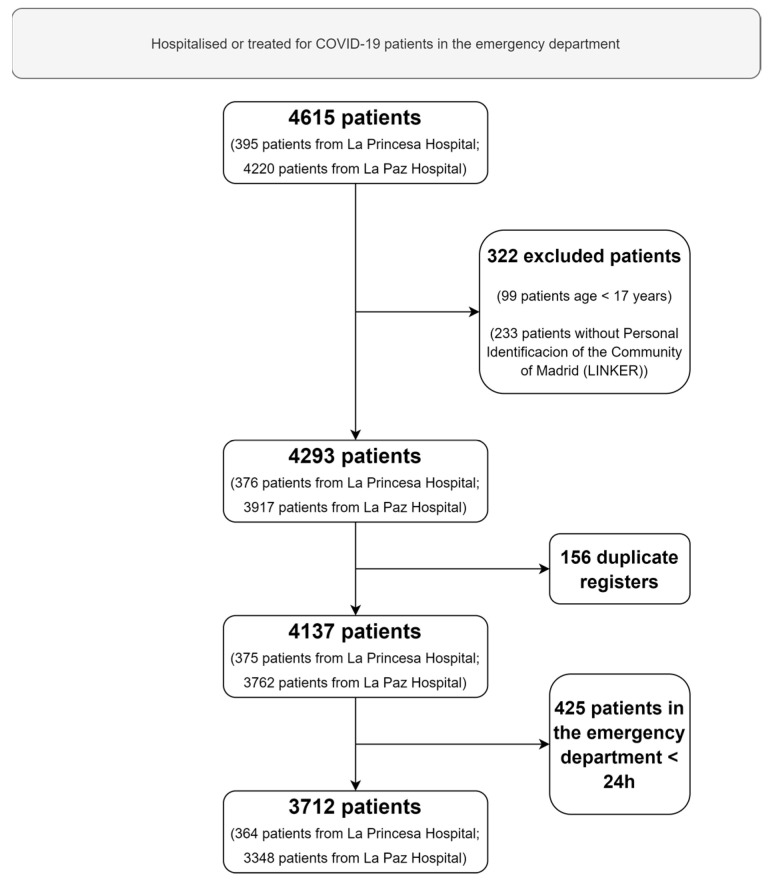
Flow chart of patients included.

A total of 3712 patients were consecutively hospitalised or treated for COVID-19 in the emergency department for more than 24 h (Figure 1). The mean age of the patients was 62 years, and 1930 (52.0%) of the patients were women. Arterial hypertension (40.7%) followed by dyslipidemia (31.5%), chronic heart disease (18.5%), and diabetes mellitus (17.7%) were the most frequent comorbidities of the patients. Of these, 687 (18.5%) patients died (cases). Table 1 shows the characteristics of patients on admission and the complications during hospitalisation. 

#### 2.1.2. Propensity Score Matching Analysis

The 22,446 medicine trademarks used previous to admission for COVID-19 infection in the case-control, were classified according to ATC obtaining 689 final drugs, all of them were included in PSM analysis. Of these, 20 demonstrated a statistically significant difference for mortality (*p* < 0.05). Eleven of them demonstrated reduction in mortality (Table 2): enoxaparin, bemiparine, oral rehydration salts, azithromycin, cefuroxime, ipratropium bromide, mepyramine theophylline acetate, budesonide-formoterol fumarate, loratadine, colchicine, and salbutamol sulphate. Eight drugs demonstrated an increase in mortality: digoxin, folic acid, mirtazapine, linagliptin, enalapril, atorvastatin, allopurinol, and acetylsalicylic acid (Table 3).

Supplementary Tables S1–S3 show the ATC codes and the results of the PSM analysis of the final drugs, subgroups, and groups, respectively. 

## 3. Discussion

This study explores the association between previous use of medications and the mortality of patients hospitalised due to COVID-19. A positive association has been found with azithromycin, bemiparine, budesonide-formoterol fumarate, cefuroxime, colchicine, enoxaparin, ipratropium bromide, loratadine, mepyramine theophylline acetate, oral rehydration salts, and salbutamol sulphate. On the other hand, the analysis shows a risk associated with acetylsalicylic acid, allopurinol, atorvastatin, digoxin, enalapril, folic acid, linagliptin, and mirtazapine. However, the results should be interpreted with great caution as propensity score matching cannot assess and balance all the factors that come into play in the clinical management of patients and that may be present in the circumstances of the study. Thus, propensity score matching analyses may omit, due to nonrecognition, the effects of several clinically significant, but not considered, factors that can affect the outcomes of the analyses being reported, causing them to possibly be misleading, or at best hypothesis-generating [17]. However, a drug-morbidity interaction analysis has been performed in the propensity score matching. The results demonstrated an interaction effect in the propensity score analysis that could explain the association of its use with the risk of death from COVID-19 disease.

### 3.1. Final Substances Significantly Associated with Mortality

#### 3.1.1. Digoxin

Atrial arrhythmias are common among hospitalised COVID-19 patients and are independently associated with increased mortality [18]. Several in vitro and in vivo studies have discovered the antiviral and anti-inflammatory properties of digoxin by inhibiting the entry of the coronavirus into cells and suppressing the cytokine storm [19,20]. However, in 2015, three independent meta-analyses raised concerns about the association of digoxin treatment with an increased risk of mortality in patients with atrial fibrillation (AF) and heart failure (HF) [21,22,23]. A 2019 update confirmed that the use of digoxin is associated with higher mortality in patients with AF or HF [24]. These data are consistent with the risk results found in this study could be due to the interaction with chronic heart disease.

#### 3.1.2. Folic Acid

Systemic inflammation, immune system impairment, and sarcopenia could act as crucial factors linking nutritional status and the course and outcome of COVID-19 [25]. Current data suggest that pregnant women have similar disease course and outcomes compared to nonpregnant people; however, pregnant women may have increased risk of hospitalisation and intensive care unit (ICU) admission. Among patients who develop severe and critical disease, major maternal morbidity and mortality have been described including cardiomyopathy, mechanical ventilation, extracorporeal membrane oxygenation, and death [26]. These data are consistent with the risk results found in this study and could be due to the interaction with malnutrition and pregnancy.

#### 3.1.3. Mirtazapine

We cannot explain the risk associated with mirtazapine outside of the risk of the sedative effects of this drug, especially in comorbid elderly patients [27,28,29]. Nevertheless, patients with mental illness are at high risk for SARS-CoV-2 infection and COVID-19-related death. Behavioural changes associated with cognitive deterioration increase the SARS-CoV-2 infection risk, and severe medical conditions and delayed treatment increase the COVID-19-related mortality risk in patients with mental illness [30].

#### 3.1.4. Linagliptin

Inflammation is implicated in the development and severity of COVID-19 infection, as well as in the pathophysiology of diabetes. Diabetes is also recognised as a considerable risk factor for COVID-19 morbidity and mortality. Furthermore, some inflammatory markers [i.e., C-reactive protein (CRP), interleukin-6 (IL-6), and ferritin] were reported as solid predictors of worse outcomes in COVID-19 positive patients. The same biomarkers have been associated with poor glycemic control. However, linagliptin can cause side effects, such as acidosis, dehydration, kidney problems, hypoglycemia, and increased cholesterol in the blood [31]. Chronic kidney disease, associated with linagliptin in the PSM interaction analysis, seems to be associated with enhanced risk of COVID-19 mortality. The disease course and outcomes in COVID-19 patients are associated with baseline estimated glomerular filtration rate [32]. 

#### 3.1.5. Enalapril

There is some evidence from retrospective trials suggesting that using an ACEI or an ARB in patients with hypertension who were hospitalised for COVID-19 was associated with similar or lower mortality rates, as compared with patients who were not taking a drug from either class prior to infection [33]. A systematic review of observational studies and trials until 4 May 2020 that examined the association and effects of ACEIs or ARBs on risk for SARS-CoV-2 infection and COVID-19 disease severity in adults found high-certainty evidence, suggesting that ACEI or ARB use is not associated with more severe COVID-19 disease [34]. A critical confounder in retrospective studies was revealed in data on patients with COVID-19. Approximately 50% of the patients who had been prescribed ACE inhibitors or ARBs discontinued the medication when they were hospitalised [35]. Hypertension increases COVID-19 severity due to underlying endothelial dysfunctions and coagulopathy. COVID-19 might augment the hypertensive complications due to the down-regulation of ACE2 [36]. The benefit of using ACEIs or ARBs in the treatment of hypertensive patients with COVID-19 is now being explored in randomised clinical trials. Underlying diseases, such as chronic kidney disease associated with enalapril use, could also explain this increased risk [29].

#### 3.1.6. Atorvastatin

Several studies have observed a decrease in total cholesterol, LDL-C, and HDL-C levels in patients with COVID-19 infections. In most studies, the decrease in LDL-C and/or HDL-C was more profound with a greater severity of illness. LDL-C and HDL-C levels were inversely correlated with C-reactive protein (CRP) levels, i.e., the lower the LDL-C or HDL-C level, the higher the CRP levels. Patients with low HDL-C levels on admission to the hospital were at an increased risk of developing a severe disease compared to patients with high HDL-C levels [37]. Patients with various infections (gram-positive bacterial, gram-negative bacterial, viral, tuberculosis, and parasites) have similar alterations in plasma lipid levels. Specifically, total cholesterol, LDL-C, and HDL-C levels are decreased, while plasma triglyceride levels may be elevated or inappropriately normal for the poor nutritional status [38]. One needs to be aware that certain drugs that are used to treat COVID-19 infections may interact with lipid lowering drugs. Remdesivir is metabolised by the CYP3A4 pathway, so it would be advisable to avoid statins that are also metabolised by this pathway (atorvastatin, simvastatin, and lovastatin) [39]. With the antiretroviral drugs (lopinavir/ritonavir), the use of low dose rosuvastatin therapy is recommended [40]. Tocilizumab interferes with both the CYP3A4 and CYP2C9 pathways of metabolism, and therefore it is recommended to temporarily suspend treatment with statins [41].

#### 3.1.7. Allopurinol

A study to assess gout management during the COVID-19 pandemic through surveys demonstrated that gout flares were common: 63% had ≥1 gout flare monthly; 11% underwent emergency room/urgent care; and 2% were hospitalised with gout flares. Between 41% and 56% of respondents reported more difficulty with gout management and functional status related to COVID-19, this could explain the detrimental effect of allopurinol in this study [42]. The use of allopurinol associated with cancer treatment in this study could also explain the increased risk, as these patients are susceptible to serious clinical adverse events and increased mortality from COVID-19 infection, as well as morbidity and mortality from its underlying malignancy [43].

#### 3.1.8. Acetylsalicylic Acid

The results of a meta-analysis of three retrospective observational studies suggested that there is no protective effect of aspirin on mortality from COVID-19. Studies likely suggest that aspirin does not reduce mortality in these patients, although patients taking aspirin tended to have more risk factors for severe COVID-19 infection (e.g., advanced age, pre-existing coronary artery disease, diabetes mellitus, etc.) [44]. Morbidity appears to be a greater factor than aspirin use.

### 3.2. Final Substances Significantly Associated with Survival

#### 3.2.1. Enoxaparine and Bemiparine

SARS-CoV-2 infection has been linked to a higher risk of mortality compared to influenza, which is mainly due to severe secondary diseases, such as acute respiratory distress syndrome (ARDS). In turn, ARDS is characterised by an acute inflammation and excessive coagulation cascade activity, raising the vulnerability for venous thromboembolic events. In accordance with previous studies, our study outlines that anti-coagulation may constitute a promising tool for treating SARS-CoV-2, reducing both the cytokine storm and the risk for thrombotic complications [45].

#### 3.2.2. Oral Rehydration Salts

We do not know the mechanism for the protection of oral rehydration with salts beyond the protective effect against dehydration.

#### 3.2.3. Azithromycin

Azithromycin presents in vitro activity against SARS-CoV-2 and could act at different points of the viral cycle. Its immunomodulatory properties include the ability to downregulate cytokine production, maintain epithelial cell integrity, and prevent lung fibrosis. Azithromycin use was associated with a reduction in mortality and days on a ventilator in other viral infections. These properties could be beneficial throughout the COVID-19 infection. However, the evidence of its use is scarce and of low quality. Azithromycin has been assessed in retrospective observational studies mainly in combination with hydroxychloroquine, which has shown no benefit [46]. This macrolide presents a well-known safety profile. Upcoming clinical trials will determine the role of azithromycin in COVID-19 (including the stage of the disease where it offers the maximal benefits and the effect of its combination with other drugs).

#### 3.2.4. Cefuroxime

Cefuroxime is a second-generation cephalosporin antibiotic. It has broad-spectrum activity and is commonly used for the treatment of both upper and lower respiratory tract infections, Lyme disease, and genitourinary tract infections. Several studies have reported cefuroxime as a potential inhibitor of three essential SARS-CoV-2 proteins; main protease, RNA-dependent RNA polymerase, and ACE2-Spike complex [47,48,49]. Further in vitro and in vivo studies are required to evaluate the potential of cefuroxime for COVID-19.

#### 3.2.5. Inhaled Glucocorticoids and Bronchodilators

Multiple early reports of patients admitted to the hospital with COVID-19 demonstrated that patients with chronic respiratory disease were significantly under-represented in these cohorts. We hypothesised that the widespread use of inhaled glucocorticoids among these patients was responsible for this finding and tested if inhaled glucocorticoids would be an effective treatment for early COVID-19 [50]. An open-label, parallel-group, phase 2, randomised controlled trial (Steroids in COVID-19; STOIC) of inhaled budesonide, compared with usual care, in adults within seven days of the onset of mild COVID-19 symptoms found that early administration of inhaled budesonide reduced the likelihood of needing urgent medical care and reduced recovery time after early COVID-19. The number of patients that needed to treat with inhaled budesonide to reduce COVID-19 deterioration was eight. Clinical recovery was one day shorter in the budesonide group compared with the usual care group (median seven days [95% CI 6 to 9] in the budesonide group vs. eight days [7 to 11] in the usual care group; log-rank test *p* = 0.007). Early administration of inhaled budesonide reduced the likelihood of needing urgent medical care and reduced recovery time after early COVID-19 [51]. The positive association of ipratropium bromide, mepyramine theophylline acetate, and salbutamol could be related to the rest of maintenance asthma and chronic obstructive pulmonary disease (COPD) medicines.

#### 3.2.6. Loratadine

A study of in vitro severe acute respiratory syndrome coronavirus-2 (SARS-CoV-2) spike pseudotyped viral infection experiments indicated that histamine H1 antagonists loratadine and desloratadine could prevent the entry of the pseudotyped virus into ACE2-overexpressing HEK293T cells and demonstrated that desloratadine was more effective. Molecular docking results elucidated that loratadine and desloratadine could bind to ACE2 on the interface of the SARS-CoV-2-binding area [52].

#### 3.2.7. Colchicine

Colchicine has been observed to help reduce inflammation in several inflammatory diseases. This study aims to analyse the efficacy of colchicine administration and outcomes of COVID-19. A recent systematic review and meta-analysis on COVID-19 and colchicine treatment found in a total of eight studies with 5778 COVID-19 patients included that colchicine was associated with an improvement of outcomes of COVID-19 [OR 0.43 (95% CI 0.34–0.55), *p* < 0.00001, I^2^ = 0%, fixed-effect modelling] and its subgroup, which was comprised of reduction from severe COVID-19 [OR 0.44 (95% CI 0.31–0.63), *p* < 0.00001, I2 = 0%, fixed-effect modelling] and reduction in the mortality rate of COVID-19 [OR 0.43 (95% CI 0.32–0.58), *p* < 0.00001, I2 = 0%, fixed-effect modelling] [53]. More randomised clinical trials are still needed to confirm the results of this study.

### 3.3. Strengths and Limitations

One strength of the study is the use of computerised databases, prescription, and administration of the study patients. Lack of adherence to treatment was controlled using a prescription–administration database. However, over-the-counter use is occasionally recorded and this could lead to a misclassification of the exposure. However, in this health area, non-prescription purchases are limited to paracetamol 500 mg, ibuprofen 400 mg, antacids, lotions and creams, anti-hemorrhoids, eye drops, vitamin preparations and some mucolytics, anti-cold, and antihistamines [54]. In addition to the design limitations of the type of analysis already addressed, other limitations are the retrospective nature of the study; however, the chosen outcome, mortality, is robust and clinically relevant. This study aimed to explore the association of drug use and prescription-administration data before hospitalisation, with mortality from COVID-19 disease due to any indication of appropriate or inappropriate use; thus, the adequacy of use has not been established in the study. Most medications identified as a risk had an associated risk morbidity in the drug-morbidity interaction analysis. With respect to beneficial medications, we cannot affirm that the treatment “protects” from infection, but that it improves the outcome. We think that it is irrelevant to be able to differentiate between improvement due to treatment of symptoms (protopathic bias), or improvement due to the reduction of viral load that translates into milder symptoms.

## 4. Materials and Methods

### 4.1. Study Design and Population

The retrospective case-control from two hospitals in Madrid, Spain, La Paz University Hospital and La Princesa University Hospital, included all patients, 18 years or older, hospitalised in the wards (or emergency department) with a diagnosis of COVID-19 and who either died or were discharged, from March to May 2020. Patients discharged from the emergency department after less than 24 h were not considered hospitalised and were not included in this analysis. Deaths were categorised as cases and survivors as controls. This study was approved by the Research Ethics Committee (PI-4072), by the Spanish Agency of Medicines and Medical Devices (HUL-AIN-2020-01), and registered in the EU PAS Register (EUPAS34331). Informed consent was not sought due to the emergency situation and because the data collection was retrospective. The program complied with the Spanish Personal Data Protection Law [55]. 

COVID-19 infection was diagnosed by a positive reverse transcription-polymerase chain reaction (RT-PCR) obtained from nasopharyngeal and oropharyngeal specimens. RT-PCR was determined using a commercial and in-house method in the microbiology department of the hospitals. Case inclusion criteria were hospitalisation for COVID-19 infection and being at least 18 years of age.

### 4.2. Clinical Data Collection

An electronic case record form (eCRF) was used to collect hospital emergency ward patients with suspected or confirmed cases of COVID-19. It was a modified version of the WHO/International Severe Acute Respiratory and Emerging Infection Consortium CRF for severe acute respiratory infections [56]. The eCRF includes 386 variables grouped into demographic, medical history, infection-exposure history, clinical symptoms, complications, evolution, medications, laboratory results, and imaging features. The results of the first 2226 adult patients of the cohort were published elsewhere [57]. These data were collected from electronic medical records (DXC-HCIS HCIS3_10_6_st30_SERMAS_V1_HULP_P9—Healthcare Information System) by a volunteer team of resident doctors and senior medical students. Laboratory results were automatically extracted from the Integrated Laboratory System (LABTrack; TrackHealth, Woolloomooloo, Australia) of the hospitals. Real-time RT-PCR results were automatically extracted from the Laboratory Information System (Microb Dynamic; Spain) of the microbiology Departments. Medications were automatically extracted from the electronic prescription systems (EPSs) from primary care and confirmed with the electronic dispensation system (MUP; General Directorate for Healthcare Coordination of the Madrid Health Service, Madrid, Spain). All the CRFs were monitored and the data curated in the Central Clinical Research Unit (UCICEC), Service of Clinical Pharmacology of La Paz University Hospital, and validated by the study’s scientific committee. The medications were coded using the World Health Organization’s Anatomical Therapeutic Chemical (ATC) classifications [58]. The code of the drug bank and the link were also added to the final substances [59].

### 4.3. Variables and Exposure

The principal independent variable was exposure to medication in the last month previous to hospitalisation due to COVID-19 infection. The medication under evaluation was the drug the patient was exposed to until admission for COVID-19 infection. Exposure to a drug was defined as the patient’s current prescription until just before hospital admission, with dispensing verified in the period. The dependent variables were age, sex, and number of morbidities (chronic heart disease, arterial hypertension, chronic obstructive pulmonary disease, asthma, other chronic lung diseases, chronic kidney disease, chronic neurological disease, solid malignant disease, chronic hematological disease, obesity, diabetes mellitus, rheumatological disease, dementia, dyslipemia, malnutrition, and severe mental illness). 

### 4.4. Analysis

A case-control propensity score matching (PSM) analysis was performed to estimate the effect of the medication used previous to admission on the mortality of patients hospitalised for COVID-19. Confounding variables were considered to be age, sex, and the number of comorbidities. The odds ratio adjusted for covariables was estimated using a multiple logistic regression model. PSM analysis of the chemical pharmacological subgroup and the pharmacological therapeutic subgroup of ATC classification was also performed. The interactions of statistical significant final drugs with the underlying pathologies were also considered in the PSM analysis. Statistical calculations were performed under the statistical environment R, using RStudio editor (version 3.4.0) [60,61].

## 5. Conclusions

In conclusion, this study provides an exploratory association of the previous use of medications with the mortality of patients hospitalised due to COVID-19. Medication associated with survival (anticoagulants, antihistamines, azithromycin, bronchodilators, cefuroxime, colchicine, and inhaled corticosteroids) may be candidates for future clinical trials. Drugs associated with mortality show an interaction with the underlying conditions.

## Figures and Tables

**Table 1:** Characteristics on admission and complications during hospitalisation.

	[ALL] *n* = 3712 *n* (%)	Case (Deceased) *n* = 687 *n* (%)	Control (Live Discharges) *n* = 3025 *n* (%)	OR	p.ratio	p.overall
Sex						<0.001
Man	1777 (47.9%)	419 (61.0%)	1358 (44.9%)	Ref.	Ref.	
Woman	1930 (52.0%)	268 (39.0%)	1662 (54.9%)	0.52 [0.44;0.62]	<0.001	
‘Missing’	5 (0.13%)	0 (0.00%)	5 (0.17%)	0.00 [0.00;3.55]	0.261	
Age, years (mean (SD))	62.0 [48.0;78.0]	83.0 [75.0;88.0]	57.0 [44.0;72.0]	1.10 [1.09;1.11]	<0.001	<0.001
Arterial hypertension						<0.001
No	2190 (59.0%)	223 (32.5%)	1967 (65.0%)	Ref.	Ref.	
Yes	1511 (40.7%)	462 (67.2%)	1049 (34.7%)	3.88 [3.25;4.66]	<0.001	
‘Missing’	11 (0.30%)	2 (0.29%)	9 (0.30%)	1.96 [0.20;9.55]	0.406	
Diabetes mellitus						<0.001
No	3043 (82.0%)	459 (66.8%)	2584 (85.4%)	Ref.	Ref.	
Yes	656 (17.7%)	225 (32.8%)	431 (14.2%)	2.94 [2.42;3.56]	<0.001	
‘Missing’	13 (0.35%)	3 (0.44%)	10 (0.33%)	1.69 [0.30;6.59]	0.433	
Non-complicated diabetes mellitus						<0.001
No	3225 (86.9%)	536 (78.0%)	2689 (88.9%)	Ref.	Ref.	
Yes	421 (11.3%)	134 (19.5%)	287 (9.49%)	2.34 [1.85;2.95]	<0.001	
‘Missing’	66 (1.78%)	17 (2.47%)	49 (1.62%)	1.74 [0.93;3.10]	0.062	
Complicated diabetes mellitus						<0.001
No	3545 (95.5%)	623 (90.7%)	2922 (96.6%)	Ref.	Ref.	
Yes	100 (2.69%)	45 (6.55%)	55 (1.82%)	3.84 [2.50;5.85]	<0.001	
‘Missing’	67 (1.80%)	19 (2.77%)	48 (1.59%)	1.86 [1.02;3.24]	0.031	
Dislipemia						<0.001
No	2526 (68.0%)	334 (48.6%)	2192 (72.5%)	Ref.	Ref.	
Yes	1168 (31.5%)	350 (50.9%)	818 (27.0%)	2.81 [2.36;3.34]	<0.001	
‘Missing’	18 (0.48%)	3 (0.44%)	15 (0.50%)	1.31 [0.24;4.67]	0.641	
Obesity						0.001
No	3190 (85.9%)	564 (82.1%)	2626 (86.8%)	Ref.	Ref.	
Yes	459 (12.4%)	102 (14.8%)	357 (11.8%)	1.33 [1.04;1.70]	0.021	
‘Missing’	63 (1.70%)	21 (3.06%)	42 (1.39%)	2.33 [1.30;4.06]	0.003	
Chronic heart disease						<0.001
No	3007 (81.0%)	399 (58.1%)	2608 (86.2%)	Ref.	Ref.	
Yes	686 (18.5%)	283 (41.2%)	403 (13.3%)	4.59 [3.80;5.54]	<0.001	
‘Missing’	19 (0.51%)	5 (0.73%)	14 (0.46%)	2.33 [0.65;6.90]	0.130	
Chronic lung disease						<0.001
No	3475 (93.6%)	619 (90.1%)	2856 (94.4%)	Ref.	Ref.	
Yes	216 (5.82%)	60 (8.73%)	156 (5.16%)	1.77 [1.28;2.44]	<0.001	
‘Missing’	21 (0.57%)	8 (1.16%)	13 (0.43%)	2.84 [1.02;7.42]	0.030	
Chronic obstructive pulmonary disease						<0.001
No	3434 (92.5%)	588 (85.6%)	2846 (94.1%)	Ref.	Ref.	
Yes	260 (7.00%)	95 (13.8%)	165 (5.45%)	2.79 [2.11;3.67]	<0.001	
‘Missing’	18 (0.48%)	4 (0.58%)	14 (0.46%)	1.38 [0.33;4.42]	0.556	
Asthma						0.040
No	3489 (94.0%)	656 (95.5%)	2833 (93.7%)	Ref.	Ref.	
Yes	205 (5.52%)	26 (3.78%)	179 (5.92%)	0.63 [0.40;0.96]	0.024	
‘Missing’	18 (0.48%)	5 (0.73%)	13 (0.43%)	1.66 [0.46;4.99]	0.347	
Neurological chronic disease						<0.001
No	3331 (89.7%)	543 (79.0%)	2788 (92.2%)	Ref.	Ref.	
Yes	363 (9.78%)	139 (20.2%)	224 (7.40%)	3.18 [2.51;4.03]	<0.001	
‘Missing’	18 (0.48%)	5 (0.73%)	13 (0.43%)	1.97 [0.55;5.93]	0.218	
Mild liver disease						0.342
No	3601 (97.0%)	663 (96.5%)	2938 (97.1%)	Ref.	Ref.	
Yes	95 (2.56%)	19 (2.77%)	76 (2.51%)	1.11 [0.63;1.87]	0.680	
‘Missing’	16 (0.43%)	5 (0.73%)	11 (0.36%)	2.01 [0.55;6.31]	0.216	
Moderate or severe liver disease						0.043
No	3662 (98.7%)	671 (97.7%)	2991 (98.9%)	Ref.	Ref.	
Yes	35 (0.94%)	11 (1.60%)	24 (0.79%)	2.04 [0.90;4.36]	0.063	
‘Missing’	15 (0.40%)	5 (0.73%)	10 (0.33%)	2.23 [0.60;7.18]	0.167	
Chronic kidney disease						<0.001
No	3435 (92.5%)	549 (79.9%)	2886 (95.4%)	Ref.	Ref.	
Yes	260 (7.00%)	134 (19.5%)	126 (4.17%)	5.59 [4.27;7.31]	<0.001	
‘Missing’	17 (0.46%)	4 (0.58%)	13 (0.43%)	1.62 [0.38;5.26]	0.407	
GF < 30:						<0.001
No	137 (3.69%)	65 (9.46%)	72 (2.38%)	Ref.	Ref.	
Yes	104 (2.80%)	57 (8.30%)	47 (1.55%)	1.34 [0.78;2.31]	0.261	
‘Missing’	3471 (93.5%)	565 (82.2%)	2906 (96.1%)	0.22 [0.15;0.31]	<0.001	
Solid malignant disease						<0.001
No	3304 (89.0%)	546 (79.5%)	2758 (91.2%)	Ref.	Ref.	
Yes	387 (10.4%)	135 (19.7%)	252 (8.33%)	2.71 [2.14;3.42]	<0.001	
‘Missing’	21 (0.57%)	6 (0.87%)	15 (0.50%)	2.02 [0.64;5.54]	0.167	
Haematological chronic disease						<0.001
No	3481 (93.8%)	615 (89.5%)	2866 (94.7%)	Ref.	Ref.	
Yes	211 (5.68%)	67 (9.75%)	144 (4.76%)	2.17 [1.58;2.96]	<0.001	
‘Missing’	20 (0.54%)	5 (0.73%)	15 (0.50%)	1.55 [0.44;4.52]	0.400	
Rheumatological disease						<0.001
No	3284 (88.5%)	569 (82.8%)	2715 (89.8%)	Ref.	Ref.	
Yes	412 (11.1%)	113 (16.4%)	299 (9.88%)	1.80 [1.41;2.29]	<0.001	
‘Missing’	16 (0.43%)	5 (0.73%)	11 (0.36%)	2.17 [0.59;6.80]	0.175	
HIV infection						0.668
No	3671 (98.9%)	678 (98.7%)	2993 (98.9%)	Ref.	Ref.	
Yes	21 (0.57%)	4 (0.58%)	17 (0.56%)	1.04 [0.25;3.20]	0.905	
‘Missing’	20 (0.54%)	5 (0.73%)	15 (0.50%)	1.47 [0.42;4.28]	0.455	
Malnutrition						0.002
No	3670 (98.9%)	671 (97.7%)	2999 (99.1%)	Ref.	Ref.	
Yes	15 (0.40%)	8 (1.16%)	7 (0.23%)	5.10 [1.61;16.6]	0.003	
‘Missing’	27 (0.73%)	8 (1.16%)	19 (0.63%)	1.88 [0.71;4.52]	0.152	
Dementia						<0.001
No	3482 (93.8%)	565 (82.2%)	2917 (96.4%)	Ref.	Ref.	
Yes	212 (5.71%)	118 (17.2%)	94 (3.11%)	6.48 [4.82;8.72]	<0.001	
‘Missing’	18 (0.48%)	4 (0.58%)	14 (0.46%)	1.47 [0.35;4.72]	0.489	
Mental illness						<0.001
No	3327 (89.6%)	587 (85.4%)	2740 (90.6%)	Ref.	Ref.	
Yes	365 (9.83%)	96 (14.0%)	269 (8.89%)	1.67 [1.28;2.15]	<0.001	
‘Missing’	20 (0.54%)	4 (0.58%)	16 (0.53%)	1.17 [0.28;3.63]	0.752	
Non-severe mental illness, type						0.001
1	224 (6.03%)	62 (9.02%)	162 (5.36%)	Ref.	Ref.	
2	110 (2.96%)	23 (3.35%)	87 (2.88%)	0.69 [0.38;1.22]	0.184	
3	25 (0.67%)	8 (1.16%)	17 (0.56%)	1.23 [0.44;3.19]	0.643	
‘Missing’	3353 (90.3%)	594 (86.5%)	2759 (91.2%)	0.56 [0.41;0.78]	<0.001	
Severe mental illness						0.288
No	3561 (95.9%)	663 (96.5%)	2898 (95.8%)	Ref.	Ref.	
Yes	132 (3.56%)	19 (2.77%)	113 (3.74%)	0.74 [0.42;1.21]	0.218	
‘Missing’	19 (0.51%)	5 (0.73%)	14 (0.46%)	1.56 [0.44;4.61]	0.399	
Severe mental illness, type						0.017
1	74 (1.99%)	5 (0.73%)	69 (2.28%)	Ref.	Ref.	
2	22 (0.59%)	4 (0.58%)	18 (0.60%)	3.02 [0.54;15.7]	0.146	
3	36 (0.97%)	10 (1.46%)	26 (0.86%)	5.21 [1.46;21.4]	0.005	
‘Missing’	3580 (96.4%)	668 (97.2%)	2912 (96.3%)	3.17 [1.29;10.1]	0.005	
Charlson Comorbidity Index	2.00 [0.00;5.00]	5.00 [4.00;7.00]	2.00 [0.00;4.00]	1.57 [1.51;1.63]	<0.001	<0.001

GF < 30, glomerular filtration lower than 30 mL/min; HIV, human immunodeficiency virus; ICU, intensive care unit; IQR, interquartile range; NR, normal range; OR, odds ratio; p.ratio, *p*-value in the logistic regression; p.overall, p ratio of t.

**Table 2:** Final drug PSM analysis. Lower and upper limits of the 95% confidence interval for the odds ratio. The final substances significantly associated with survival.

Final Drug	ATC Code	Drug *p*-Value	OR	Lower Limit 95% CI	Upper Limit 95% CI	Power
ENOXAPARINE	B01AB05	<0.001	0.11	0.06	0.21	<0.001
BEMIPARINE	B01AB12	<0.001	0.18	0.08	0.37	0.585
ORAL REHYDRATION SALTS (GLUCOSE, POTASSIUM CHLORIDE, SODIUM CHLORIDE, TRISODIUM CITRATE)	A07CA91	<0.001	0.15	0.03	0.53	0.591
AZITHROMYCIN	J01FA10	0.002	0.46	0.26	0.78	0.517
CEFUROXIME	J01DC02	0.011	0.26	0.06	0.83	0.553
IPRATROPIUM BROMIDE	R03BB01	0.006	0.48	0.27	0.84	0.516
MEPYRAMINE THEOPHYLLINE ACETATE	R03DA12	0.015	0.00	0.00	0.85	0.853
BUDESONIDE, FORMOTEROL FUMARATE	R03AK07	0.013	0.51	0.28	0.90	0.514
LORATADINE	R06AX13	0.022	0.20	0.02	0.93	0.576
COLCHICINE	M04AC01	0.022	0.20	0.02	0.93	0.576
SALBUTAMOL SULPHATE	R03AC02	0.039	0.62	0.37	0.99	0.508

OR: odds ratio; *p*-value: statistical significance.

**Table 3:** Final drug PSM analysis. Lower and upper limits of the 95% confidence interval for the odds ratio. The final substances significantly associated with mortality.

Final Drug	ATC Code	Drug *p*-Value	OR	Lower Limit 95% CI	Upper Limit 95% CI	Power	Interactions *p* < 0.05
DIGOXIN	C01AA05	0.011	3.81	1.20	15.84	0.553	Chronic heart disease
FOLIC ACID	B03BB01	0.001	2.32	1.36	4.08	0.520	Malnutrition Pregnancy
MIRTAZAPINE	N06AX11	0.001	2.17	1.32	3.65	0.517	Mental illness
LINAGLIPTIN	A10BH05	0.025	2.12	1.05	4.52	0.519	Chronic kidney disease
ENALAPRIL	C09BA02	0.012	1.93	1.12	3.39	0.513	Chronic kidney disease
ATORVASTATIN	C10AA05	0.002	1.52	1.16	2.01	0.505	Dislipemia
ALLOPURINOL	M04AA01	0.030	1.42	1.02	1.99	0.504	Solid malignant disease
ACETYLSALICYLIC ACID	B01AC06	0.038	1.31	1.01	1.71	0.502	Chronic heart disease Diabetes, dislipemia, obesity

OR: odds ratio; *p*-value: statistical significance.

## Data Availability

This project is a sub-analysis of the COVID@HULP cohort (DOI: 10.3390/jcm9061733). The data presented in this study are available on request from the corresponding author. The data are not publicly available due to this is a sub-analysis of a cohort of patients containing information that could compromise the privacy of research participants.

## Supplementary Materials

The following are available online at https://www.mdpi.com/article/10.3390/ph15010078/s1. Table S1: Propensity score matching analysis of other final drugs. Lower and upper limits of the 95% confidence interval for the odds ratio, Table S2: Propensity score matching the analysis of the subgroup of ATC. Lower and upper limits of the 95% confidence interval for the odds ratio, Table S3: Propensity score matching analysis of the main therapeutic group. Lower and upper limits of the 95% confidence interval for the odds ratio, Complete list of the members of the COVID@HULP Working Group and other collaborators from Hospital Universitario de la Princesa.
