# Supplementary material for: A Case-Control of Patients with COVID-19 to Explore the Association of Previous Hospitalisation Use of Medication on the Mortality of COVID-19 Disease: A Propensity Score Matching Analysis
Source: Pharmaceuticals (Basel). 2022 Jan 8;15(1):78. doi: 10.3390/ph15010078 (PMC8780256; doi:10.3390/ph15010078)
Supplement: Supplementary file 1 [file pharmaceuticals-15-00078-s001.zip › Supplementary Tables.pdf]

Title: A case-control of patients with COVID-19 to explore the association of previous hospitalisation use of medication on the mortality of COVID-19 disease: a Propensity score matching analysis

Supplementary tables

Table S1 Propensity score matching analysis of other final drugs. Lower and upper limits of the 95% confidence interval for the Odds Ratio.

ATC CODE	DRUG P.VALUE	OR	LOWER LIMIT 95% CI	UPPER LIMIT 95% CI	POWER
**A01AB09**	1.000	1.00	0.01	78.56	1.000
**A01AC53**	0.250	0.00	0.00	5.32	0.803
**A02AD03**	0.500	Inf	0.03	Inf	0.789
**A02AH**	0.500	0.00	0.00	39.00	0.789
**A02AH91**	NA	NA	NA	NA	NA
**A02BA02**	0.250	Inf	0.19	Inf	0.803
**A02BA03**	0.790	0.86	0.24	2.99	0.797
**A02BC**	0.688	1.19	0.49	2.94	0.712
**A02BC01**	0.544	1.07	0.86	1.34	0.613
**A02BC02**	0.579	1.11	0.76	1.63	0.634
**A02BC03**	0.803	0.87	0.27	2.77	0.809
**A02BC04**	1.000	1.00	0.01	78.56	1.000
**A02BC05**	0.420	1.41	0.58	3.57	0.560
**A02BD08**	NA	NA	NA	NA	NA
**A02BX**	NA	NA	NA	NA	NA
**A02BX91**	1.000	1.00	0.01	78.56	1.000
**A03AA04**	0.452	2.00	0.29	22.23	0.588
**A03AA05**	0.500	0.00	0.00	39.00	0.789
**A03AB06**	0.500	0.00	0.00	39.00	0.789
**A03AX04**	0.250	Inf	0.19	Inf	0.803
**A03AX13**	0.500	Inf	0.03	Inf	0.789
**A03BB01**	0.124	0.20	0.00	1.79	0.585
**A03FA**	0.625	2.00	0.10	118.26	0.680
**A03FA01**	0.603	1.21	0.57	2.59	0.651
**A03FA03**	0.627	0.78	0.24	2.36	0.668
**A03FA06**	0.831	1.10	0.42	2.91	0.835
**A03FA08**	0.342	0.50	0.08	2.34	0.552
**A03FA51**	NA	NA	NA	NA	NA
**A04AD**	0.753	0.80	0.16	3.73	0.766
**A05AA02**	0.048	0.33	0.08	1.09	0.541
**A06AA01**	0.374	0.33	0.01	4.15	0.586
**A06AB02**	0.500	0.00	0.00	39.00	0.789
**A06AB06**	0.500	0.00	0.00	39.00	0.789
**A06AC01**	0.547	0.66	0.14	2.82	0.622
**A06AD11**	0.605	1.34	0.40	4.70	0.654
**A06AD12**	0.250	0.00	0.00	5.32	0.803
**A06AD15**	1.000	1.00	0.01	78.56	1.000
**A06AD65**	0.708	0.86	0.38	1.96	0.728

**A06AG01**	0.500	0.00	0.00	39.00	0.789
**A06AG11**	0.500	0.00	0.00	39.00	0.789
**A06AX04**	NA	NA	NA	NA	NA
**A07AA02**	0.478	0.70	0.22	2.04	0.585
**A07AA11**	0.625	2.00	0.10	118.26	0.680
**A07DA03**	1.000	1.00	0.01	78.56	1.000
**A07EA06**	NA	NA	NA	NA	NA
**A07EA07**	NA	NA	NA	NA	NA
**A07EC01**	0.250	Inf	0.19	Inf	0.803
**A07EC02**	0.452	0.50	0.04	3.49	0.588
**A07FA02**	NA	NA	NA	NA	NA
**A07XA04**	1.000	1.00	0.01	78.56	1.000
**A09AA02**	1.000	1.00	0.13	7.49	1.000
**A10AB01**	0.374	3.01	0.24	158.05	0.586
**A10AB04**	0.726	1.34	0.22	9.15	0.744
**A10AB05**	0.164	2.02	0.70	6.59	0.524
**A10AB06**	0.178	2.27	0.63	10.12	0.532
**A10AC01**	0.500	0.00	0.00	39.00	0.789
**A10AD01**	0.500	Inf	0.03	Inf	0.789
**A10AD04**	0.625	2.00	0.10	118.26	0.680
**A10AD05**	1.000	1.00	0.07	13.83	1.000
**A10AE04**	0.210	1.34	0.83	2.17	0.510
**A10AE05**	0.753	0.80	0.16	3.73	0.766
**A10AE06**	0.625	0.50	0.01	9.62	0.680
**A10BA02**	0.304	0.82	0.56	1.21	0.522
**A10BB**	0.500	0.00	0.00	39.00	0.789
**A10BB01**	0.500	0.00	0.00	39.00	0.789
**A10BB09**	0.038	4.54	0.94	43.34	0.569
**A10BB12**	1.000	1.00	0.13	7.49	1.000
**A10BD07**	1.000	1.00	0.41	2.45	1.000
**A10BD08**	0.881	0.96	0.50	1.81	0.882
**A10BD10**	0.500	0.00	0.00	39.00	0.789
**A10BD11**	1.000	1.00	0.07	13.83	1.000
**A10BD13**	0.500	0.00	0.00	39.00	0.789
**A10BD15**	0.250	Inf	0.19	Inf	0.803
**A10BD16**	NA	NA	NA	NA	NA
**A10BD20**	0.124	5.02	0.56	237.95	0.585
**A10BF01**	NA	NA	NA	NA	NA
**A10BH01**	1.000	1.00	0.27	3.76	1.000
**A10BH02**	0.141	0.45	0.12	1.42	0.528
**A10BH03**	0.500	Inf	0.03	Inf	0.789
**A10BH04**	0.250	0.00	0.00	5.32	0.803
**A10BJ01**	NA	NA	NA	NA	NA
**A10BJ02**	1.000	1.00	0.01	78.56	1.000
**A10BJ03**	0.500	0.00	0.00	39.00	0.789
**A10BJ05**	0.625	2.00	0.10	118.26	0.680

**A10BJ06**	NA	NA	NA	NA	NA
**A10BK01**	0.225	2.35	0.53	14.12	0.539
**A10BK02**	0.500	Inf	0.03	Inf	0.789
**A10BK03**	0.386	1.76	0.44	8.22	0.557
**A10BX02**	1.000	1.00	0.42	2.36	1.000
**A11CA01**	0.250	Inf	0.19	Inf	0.803
**A11CC04**	0.625	0.50	0.01	9.62	0.680
**A11CC05**	0.226	1.68	0.68	4.39	0.520
**A11CC06**	0.809	0.97	0.76	1.24	0.814
**A11DA01**	0.625	2.00	0.10	118.26	0.680
**A11DB**	0.250	0.00	0.00	5.32	0.803
**A11DB91**	NA	NA	NA	NA	NA
**A11EX**	NA	NA	NA	NA	NA
**A11GA01**	0.500	0.00	0.00	39.00	0.789
**A11HA03**	0.374	3.01	0.24	158.05	0.586
**A11HA05**	0.250	0.00	0.00	5.32	0.803
**A12AA04**	0.141	0.45	0.12	1.42	0.528
**A12AA92**	0.250	Inf	0.19	Inf	0.803
**A12AX**	0.205	0.74	0.46	1.20	0.510
**A12AX93**	0.823	1.05	0.66	1.68	0.827
**A12AX96**	0.039	7.05	0.90	318.27	0.604
**A12BA01**	0.687	1.50	0.17	18.03	0.716
**A12BA04**	0.687	1.50	0.17	18.03	0.716
**A12BA30**	NA	NA	NA	NA	NA
**A12CC30**	0.687	0.67	0.06	5.83	0.716
**A12CX91**	0.250	Inf	0.19	Inf	0.803
**A15ZZ91**	0.500	Inf	0.03	Inf	0.789
**B01AA03**	0.250	Inf	0.19	Inf	0.803
**B01AA07**	0.133	1.33	0.90	1.97	0.505
**B01AB11**	NA	NA	NA	NA	NA
**B01AC04**	0.870	0.95	0.46	1.92	0.871
**B01AC05**	NA	NA	NA	NA	NA
**B01AC18**	0.452	0.50	0.04	3.49	0.588
**B01AC22**	NA	NA	NA	NA	NA
**B01AC23**	0.250	Inf	0.19	Inf	0.803
**B01AC24**	0.125	0.00	0.00	2.42	0.820
**B01AD01**	0.500	0.00	0.00	39.00	0.789
**B01AE07**	1.000	1.00	0.27	3.76	1.000
**B01AF01**	0.158	1.69	0.78	3.82	0.514
**B01AF02**	0.576	0.85	0.47	1.54	0.633
**B01AF03**	0.605	0.75	0.21	2.47	0.654
**B01AX05**	NA	NA	NA	NA	NA
**B02AA02**	0.500	Inf	0.03	Inf	0.789
**B03AA01**	0.044	2.04	0.97	4.51	0.518
**B03AA07**	0.897	0.97	0.56	1.67	0.898
**B03AA91**	NA	NA	NA	NA	NA

**B03AB**	0.179	3.02	0.54	30.65	0.551
**B03AB09**	0.422	0.62	0.16	2.17	0.565
**B03AB91**	0.500	0.00	0.00	39.00	0.789
**B03AB92**	1.000	1.00	0.01	78.56	1.000
**B03BA01**	0.110	1.49	0.89	2.54	0.507
**B03BA51**	0.579	0.71	0.18	2.62	0.639
**B05BB91**	0.250	0.00	0.00	5.32	0.803
**C01BC03**	NA	NA	NA	NA	NA
**C01BC04**	1.000	1.00	0.13	7.49	1.000
**C01BD01**	1.000	1.00	0.13	7.49	1.000
**C01BD07**	1.000	1.00	0.01	78.56	1.000
**C01CA24**	0.500	Inf	0.03	Inf	0.789
**C01DA02**	0.876	1.05	0.54	2.07	0.877
**C01DA14**	0.178	2.27	0.63	10.12	0.532
**C01DA52**	0.866	1.06	0.51	2.21	0.868
**C01EB15**	0.500	0.00	0.00	39.00	0.789
**C01EB17**	0.031	Inf	0.92	Inf	0.844
**C01EB18**	1.000	1.00	0.35	2.86	1.000
**C02AC05**	0.500	Inf	0.03	Inf	0.789
**C02CA04**	0.177	1.52	0.80	2.96	0.511
**C02DB02**	0.500	0.00	0.00	39.00	0.789
**C02DC01**	NA	NA	NA	NA	NA
**C03AA03**	0.698	1.17	0.50	2.79	0.720
**C03BA04**	0.288	2.51	0.41	26.45	0.554
**C03BA11**	0.823	0.90	0.32	2.48	0.827
**C03CA01**	0.142	1.24	0.92	1.68	0.504
**C03CA04**	1.000	1.00	0.51	1.98	1.000
**C03DA01**	0.545	1.21	0.63	2.33	0.615
**C03DA04**	0.688	1.19	0.49	2.94	0.712
**C03EA01**	0.646	1.25	0.44	3.68	0.681
**C03EA04**	NA	NA	NA	NA	NA
**C04AD03**	0.208	2.01	0.62	7.55	0.528
**C05AA01**	NA	NA	NA	NA	NA
**C05AE01**	0.125	0.00	0.00	2.42	0.820
**C05AX03**	0.500	0.00	0.00	39.00	0.789
**C05BX01**	0.500	0.00	0.00	39.00	0.789
**C05CA04**	0.500	0.00	0.00	39.00	0.789
**C05CA53**	0.250	0.00	0.00	5.32	0.803
**C07AA05**	0.422	0.62	0.16	2.17	0.565
**C07AA07**	0.500	Inf	0.03	Inf	0.789
**C07AB02**	0.065	0.25	0.03	1.25	0.563
**C07AB03**	1.000	1.00	0.52	1.91	1.000
**C07AB07**	0.373	1.16	0.83	1.61	0.538
**C07AB12**	0.625	0.50	0.01	9.62	0.680
**C07AG02**	0.058	1.70	0.95	3.11	0.510
**C07BB07**	NA	NA	NA	NA	NA

**C07CB03**	0.250	Inf	0.19	Inf	0.803
**C08CA01**	0.931	0.98	0.69	1.41	0.931
**C08CA05**	0.625	2.00	0.10	118.26	0.680
**C08CA09**	0.500	Inf	0.03	Inf	0.789
**C08CA11**	1.000	1.00	0.19	5.39	1.000
**C08CA12**	0.288	0.40	0.04	2.44	0.554
**C08CA13**	0.813	0.89	0.30	2.61	0.819
**C08DA01**	0.687	0.67	0.06	5.83	0.716
**C08DB01**	0.386	1.76	0.44	8.22	0.557
**C09AA01**	0.062	0.00	0.00	1.51	0.833
**C09AA02**	0.370	0.87	0.64	1.19	0.537
**C09AA03**	0.813	1.13	0.38	3.38	0.819
**C09AA04**	1.000	1.00	0.13	7.49	1.000
**C09AA05**	0.753	0.80	0.16	3.73	0.766
**C09AA06**	0.500	Inf	0.03	Inf	0.789
**C09AA16**	0.218	0.25	0.01	2.53	0.577
**C09BA01**	1.000	1.00	0.01	78.56	1.000
**C09BA03**	0.342	0.50	0.08	2.34	0.552
**C09BA04**	0.250	Inf	0.19	Inf	0.803
**C09BA05**	1.000	1.00	0.07	13.83	1.000
**C09BA06**	NA	NA	NA	NA	NA
**C09BB02**	1.000	1.00	0.19	5.39	1.000
**C09BB10**	1.000	1.00	0.01	78.56	1.000
**C09CA**	0.250	0.00	0.00	5.32	0.803
**C09CA01**	0.683	1.12	0.63	2.00	0.707
**C09CA02**	0.250	Inf	0.19	Inf	0.803
**C09CA03**	0.304	1.57	0.63	4.13	0.530
**C09CA04**	0.538	1.31	0.52	3.35	0.612
**C09CA06**	1.000	1.00	0.23	4.37	1.000
**C09CA07**	0.342	0.50	0.08	2.34	0.552
**C09CA08**	0.356	0.63	0.21	1.80	0.543
**C09DA**	0.125	Inf	0.41	Inf	0.820
**C09DA01**	0.372	0.72	0.32	1.56	0.543
**C09DA02**	0.250	0.00	0.00	5.32	0.803
**C09DA03**	0.131	2.02	0.76	5.95	0.522
**C09DA04**	0.605	1.34	0.40	4.70	0.654
**C09DA06**	0.774	1.20	0.30	5.00	0.783
**C09DA07**	0.452	0.50	0.04	3.49	0.588
**C09DA08**	0.075	0.36	0.08	1.22	0.537
**C09DB01**	0.579	0.71	0.18	2.62	0.639
**C09DB02**	1.000	1.00	0.13	7.49	1.000
**C09DB04**	1.000	1.00	0.01	78.56	1.000
**C09DX01**	0.687	0.67	0.06	5.83	0.716
**C09DX03**	0.627	1.29	0.42	4.10	0.668
**C09DX04**	0.299	1.81	0.54	6.91	0.536
**C09XA02**	0.625	2.00	0.10	118.26	0.680

**C10AA01**	0.873	0.97	0.70	1.35	0.874
**C10AA02**	0.507	1.67	0.32	10.80	0.604
**C10AA03**	0.813	0.89	0.30	2.61	0.819
**C10AA04**	1.000	1.00	0.07	13.83	1.000
**C10AA07**	0.740	0.89	0.43	1.83	0.754
**C10AA08**	0.646	1.25	0.44	3.68	0.681
**C10AB02**	0.500	0.00	0.00	39.00	0.789
**C10AB04**	0.687	0.67	0.06	5.83	0.716
**C10AB05**	0.113	2.19	0.77	7.06	0.525
**C10AC01**	1.000	1.00	0.13	7.49	1.000
**C10AX06**	0.605	1.34	0.40	4.70	0.654
**C10AX09**	1.000	1.00	0.39	2.56	1.000
**C10BA02**	0.342	2.01	0.43	12.46	0.552
**C10BA03**	0.625	0.50	0.01	9.62	0.680
**C10BA05**	0.042	0.41	0.14	1.04	0.528
**C10BA06**	0.108	0.28	0.03	1.50	0.557
**C10BX03**	0.250	Inf	0.19	Inf	0.803
**C10BX06**	0.125	Inf	0.41	Inf	0.820
**D01AC01**	0.774	1.20	0.30	5.00	0.783
**D01AC08**	0.478	0.70	0.22	2.04	0.585
**D01AC10**	0.500	0.00	0.00	39.00	0.789
**D01AC11**	0.500	0.00	0.00	39.00	0.789
**D01AC12**	0.500	Inf	0.03	Inf	0.789
**D01AC14**	1.000	1.00	0.01	78.56	1.000
**D01AC52**	0.500	Inf	0.03	Inf	0.789
**D01AE14**	0.870	0.95	0.46	1.92	0.871
**D01AE15**	0.625	0.50	0.01	9.62	0.680
**D01AE16**	0.625	0.50	0.01	9.62	0.680
**D01BA02**	NA	NA	NA	NA	NA
**D03BA52**	0.374	3.01	0.24	158.05	0.586
**D05AX02**	0.500	0.00	0.00	39.00	0.789
**D05AX52**	0.034	3.71	0.97	20.78	0.554
**D05BB02**	0.250	Inf	0.19	Inf	0.803
**D05BX90**	NA	NA	NA	NA	NA
**D06AX01**	1.000	1.00	0.07	13.83	1.000
**D06AX04**	NA	NA	NA	NA	NA
**D06AX09**	0.065	0.25	0.03	1.25	0.563
**D06BA01**	0.062	Inf	0.66	Inf	0.833
**D06BB10**	NA	NA	NA	NA	NA
**D06BB12**	NA	NA	NA	NA	NA
**D06BX01**	1.000	1.00	0.01	78.56	1.000
**D07AA02**	0.625	0.50	0.01	9.62	0.680
**D07AB01**	1.000	1.00	0.01	78.56	1.000
**D07AB02**	NA	NA	NA	NA	NA
**D07AC01**	1.000	1.00	0.27	3.76	1.000
**D07AC04**	NA	NA	NA	NA	NA

**D07AC13**	0.823	0.90	0.32	2.48	0.827
**D07AC14**	0.062	0.51	0.23	1.09	0.516
**D07AC15**	0.374	3.01	0.24	158.05	0.586
**D07AC16**	0.625	0.50	0.01	9.62	0.680
**D07AC17**	0.374	3.01	0.24	158.05	0.586
**D07AC18**	0.342	0.50	0.08	2.34	0.552
**D07AD01**	0.554	0.78	0.32	1.87	0.621
**D07CA01**	NA	NA	NA	NA	NA
**D07CB01**	NA	NA	NA	NA	NA
**D07CC01**	0.500	Inf	0.03	Inf	0.789
**D07XC01**	0.625	0.50	0.01	9.62	0.680
**D09AA09**	0.500	Inf	0.03	Inf	0.789
**D10AF01**	1.000	1.00	0.01	78.56	1.000
**D10AF02**	NA	NA	NA	NA	NA
**D10BA01**	NA	NA	NA	NA	NA
**D11AH01**	1.000	1.00	0.19	5.39	1.000
**D11AH02**	0.753	0.80	0.16	3.73	0.766
**D11AX01**	NA	NA	NA	NA	NA
**D11AX10**	NA	NA	NA	NA	NA
**D11AX18**	1.000	1.00	0.19	5.39	1.000
**D11AX22**	NA	NA	NA	NA	NA
**G01AC05**	NA	NA	NA	NA	NA
**G01AF02**	0.124	0.20	0.00	1.79	0.585
**G01AF04**	NA	NA	NA	NA	NA
**G01AF12**	NA	NA	NA	NA	NA
**G01AF20**	NA	NA	NA	NA	NA
**G02BB01**	NA	NA	NA	NA	NA
**G02CB03**	NA	NA	NA	NA	NA
**G02CC03**	NA	NA	NA	NA	NA
**G03AA07**	NA	NA	NA	NA	NA
**G03AA09**	NA	NA	NA	NA	NA
**G03AA11**	NA	NA	NA	NA	NA
**G03AA12**	NA	NA	NA	NA	NA
**G03AA13**	NA	NA	NA	NA	NA
**G03AA14**	NA	NA	NA	NA	NA
**G03AA16**	NA	NA	NA	NA	NA
**G03AB08**	NA	NA	NA	NA	NA
**G03AC09**	NA	NA	NA	NA	NA
**G03AD02**	NA	NA	NA	NA	NA
**G03BA03**	0.500	Inf	0.03	Inf	0.789
**G03CA03**	NA	NA	NA	NA	NA
**G03CA04**	NA	NA	NA	NA	NA
**G03CA09**	NA	NA	NA	NA	NA
**G03CX01**	NA	NA	NA	NA	NA
**G03DA02**	NA	NA	NA	NA	NA
**G03DA04**	NA	NA	NA	NA	NA

**G03DC02**	NA	NA	NA	NA	NA
**G03FB01**	NA	NA	NA	NA	NA
**G03HA01**	0.500	Inf	0.03	Inf	0.789
**G03HB01**	NA	NA	NA	NA	NA
**G03XC01**	NA	NA	NA	NA	NA
**G03XC02**	NA	NA	NA	NA	NA
**G04BC**	0.500	Inf	0.03	Inf	0.789
**G04BC91**	0.500	Inf	0.03	Inf	0.789
**G04BD04**	0.500	Inf	0.03	Inf	0.789
**G04BD06**	NA	NA	NA	NA	NA
**G04BD07**	0.452	0.50	0.04	3.49	0.588
**G04BD08**	0.386	1.76	0.44	8.22	0.557
**G04BD09**	0.500	Inf	0.03	Inf	0.789
**G04BD11**	0.726	0.75	0.11	4.45	0.744
**G04BD12**	0.207	1.76	0.69	4.89	0.521
**G04BE01**	0.500	0.00	0.00	39.00	0.789
**G04BE03**	0.250	0.00	0.00	5.32	0.803
**G04BE08**	0.500	Inf	0.03	Inf	0.789
**G04BE10**	NA	NA	NA	NA	NA
**G04CA01**	0.625	2.00	0.10	118.26	0.680
**G04CA02**	0.912	0.98	0.62	1.54	0.913
**G04CA03**	0.250	0.00	0.00	5.32	0.803
**G04CA04**	0.870	1.06	0.52	2.16	0.871
**G04CA52**	0.129	1.40	0.89	2.22	0.506
**G04CA53**	0.148	1.89	0.75	5.19	0.519
**G04CB01**	1.000	1.00	0.13	7.49	1.000
**G04CB02**	0.687	1.50	0.17	18.03	0.716
**G04CX02**	0.356	1.58	0.56	4.84	0.543
**G04CX51**	0.125	Inf	0.41	Inf	0.820
**H01BA02**	NA	NA	NA	NA	NA
**H01CB02**	0.500	0.00	0.00	39.00	0.789
**H01CB03**	NA	NA	NA	NA	NA
**H02AA02**	1.000	1.00	0.01	78.56	1.000
**H02AB01**	NA	NA	NA	NA	NA
**H02AB02**	0.141	2.22	0.71	8.19	0.528
**H02AB04**	0.547	0.66	0.14	2.82	0.622
**H02AB07**	0.492	1.17	0.73	1.90	0.586
**H02AB08**	0.500	0.00	0.00	39.00	0.789
**H02AB09**	1.000	1.00	0.07	13.83	1.000
**H02AB13**	1.000	1.00	0.01	78.56	1.000
**H03AA01**	0.227	0.81	0.57	1.16	0.510
**H03BB01**	0.500	Inf	0.03	Inf	0.789
**H03BB02**	1.000	1.00	0.13	7.49	1.000
**H03CA**	NA	NA	NA	NA	NA
**H03CA91**	NA	NA	NA	NA	NA
**H04AA01**	0.726	1.34	0.22	9.15	0.744

**H05AA02**	0.625	2.00	0.10	118.26	0.680
**H05BA01**	NA	NA	NA	NA	NA
**H05BX02**	0.507	1.67	0.32	10.80	0.604
**J01AA02**	0.500	0.00	0.00	39.00	0.789
**J01AA08**	0.500	0.00	0.00	39.00	0.789
**J01CA04**	0.858	0.94	0.43	2.04	0.861
**J01CE02**	NA	NA	NA	NA	NA
**J01CF02**	0.218	0.25	0.01	2.53	0.577
**J01CR02**	0.074	0.58	0.29	1.10	0.512
**J01DB05**	0.500	0.00	0.00	39.00	0.789
**J01DD08**	0.299	0.55	0.14	1.85	0.536
**J01DD16**	0.386	1.76	0.44	8.22	0.557
**J01EE01**	0.605	1.34	0.40	4.70	0.654
**J01FA09**	0.125	0.00	0.00	2.42	0.820
**J01FF01**	1.000	1.00	0.07	13.83	1.000
**J01GB03**	0.500	0.00	0.00	39.00	0.789
**J01MA02**	0.131	0.50	0.17	1.32	0.522
**J01MA06**	1.000	1.00	0.01	78.56	1.000
**J01MA12**	0.133	0.66	0.36	1.17	0.509
**J01MA14**	0.374	0.33	0.01	4.15	0.586
**J01XE01**	0.726	1.34	0.22	9.15	0.744
**J01XX01**	0.646	0.87	0.45	1.67	0.680
**J02AC01**	0.452	0.50	0.04	3.49	0.588
**J02AC02**	0.500	0.00	0.00	39.00	0.789
**J04AB02**	NA	NA	NA	NA	NA
**J04AC51**	NA	NA	NA	NA	NA
**J05AB01**	1.000	1.00	0.13	7.49	1.000
**J05AB11**	0.625	0.50	0.01	9.62	0.680
**J05AH02**	0.500	0.00	0.00	39.00	0.789
**L01BA01**	1.000	1.00	0.01	78.56	1.000
**L01BC52**	0.625	0.50	0.01	9.62	0.680
**L01XX05**	0.625	0.50	0.01	9.62	0.680
**L02AB01**	1.000	1.00	0.01	78.56	1.000
**L02AE02**	0.145	0.37	0.06	1.56	0.540
**L02AE03**	0.500	0.00	0.00	39.00	0.789
**L02AE04**	0.753	0.80	0.16	3.73	0.766
**L02BA01**	0.500	0.00	0.00	39.00	0.789
**L02BA03**	0.500	Inf	0.03	Inf	0.789
**L02BB03**	1.000	1.00	0.07	13.83	1.000
**L02BG03**	0.250	Inf	0.19	Inf	0.803
**L02BG04**	0.452	0.50	0.04	3.49	0.588
**L02BG06**	NA	NA	NA	NA	NA
**L04AA06**	0.125	Inf	0.41	Inf	0.820
**L04AA10**	NA	NA	NA	NA	NA
**L04AA13**	0.125	0.00	0.00	2.42	0.820
**L04AA18**	0.218	4.01	0.40	197.95	0.577

**L04AD01**	0.500	0.00	0.00	39.00	0.789
**L04AD02**	0.008	Inf	1.45	Inf	0.861
**L04AX01**	0.452	2.00	0.29	22.23	0.588
**L04AX03**	0.235	1.85	0.62	6.12	0.526
**M01AB01**	NA	NA	NA	NA	NA
**M01AB05**	0.625	2.00	0.10	118.26	0.680
**M01AB16**	0.625	0.50	0.01	9.62	0.680
**M01AB55**	NA	NA	NA	NA	NA
**M01AC05**	0.500	0.00	0.00	39.00	0.789
**M01AC06**	0.500	0.00	0.00	39.00	0.789
**M01AE01**	0.314	0.74	0.40	1.37	0.527
**M01AE02**	0.547	0.66	0.14	2.82	0.622
**M01AE03**	NA	NA	NA	NA	NA
**M01AE14**	NA	NA	NA	NA	NA
**M01AE17**	0.040	0.35	0.10	1.04	0.536
**M01AE52**	0.500	0.00	0.00	39.00	0.789
**M01AG01**	NA	NA	NA	NA	NA
**M01AH01**	0.374	3.01	0.24	158.05	0.586
**M01AH05**	0.687	1.50	0.17	18.03	0.716
**M01AX02**	NA	NA	NA	NA	NA
**M01AX05**	0.250	0.00	0.00	5.32	0.803
**M01AX21**	0.500	0.00	0.00	39.00	0.789
**M01AX25**	0.075	0.36	0.08	1.22	0.537
**M01CX**	0.250	0.00	0.00	5.32	0.803
**M02AA15**	NA	NA	NA	NA	NA
**M02AA28**	0.500	0.00	0.00	39.00	0.789
**M03BA03**	0.500	Inf	0.03	Inf	0.789
**M03BX01**	0.288	0.40	0.04	2.44	0.554
**M03BX02**	0.500	Inf	0.03	Inf	0.789
**M03BX08**	0.125	0.00	0.00	2.42	0.820
**M04AA03**	0.288	2.51	0.41	26.45	0.554
**M04AC51**	0.125	Inf	0.41	Inf	0.820
**M05BA04**	0.579	0.71	0.18	2.62	0.639
**M05BA06**	1.000	1.00	0.13	7.49	1.000
**M05BA07**	0.547	1.50	0.35	7.28	0.622
**M05BB03**	0.625	2.00	0.10	118.26	0.680
**M05BX04**	0.866	1.06	0.51	2.21	0.868
**N01BA02**	NA	NA	NA	NA	NA
**N01BB02**	0.625	0.50	0.01	9.62	0.680
**N01BB20**	0.687	1.50	0.17	18.03	0.716
**N01BX04**	0.452	2.00	0.29	22.23	0.588
**N02AA01**	0.145	2.68	0.64	15.78	0.540
**N02AA05**	0.500	Inf	0.03	Inf	0.789
**N02AA55**	0.726	1.34	0.22	9.15	0.744
**N02AB03**	0.050	2.28	0.94	6.11	0.524
**N02AE01**	0.374	0.33	0.01	4.15	0.586

**N02AJ06**	0.062	0.43	0.15	1.12	0.525
**N02AJ07**	0.500	0.00	0.00	39.00	0.789
**N02AJ08**	NA	NA	NA	NA	NA
**N02AJ13**	0.200	0.77	0.51	1.16	0.508
**N02AJ14**	0.500	0.00	0.00	39.00	0.789
**N02AX02**	0.740	1.12	0.55	2.32	0.754
**N02AX06**	0.790	0.86	0.24	2.99	0.797
**N02BA01**	0.500	Inf	0.03	Inf	0.789
**N02BB**	0.288	0.40	0.04	2.44	0.554
**N02BB02**	0.806	1.03	0.80	1.32	0.812
**N02BE01**	0.448	1.09	0.87	1.35	0.565
**N02BG91**	0.500	0.00	0.00	39.00	0.789
**N02CA52**	0.625	0.50	0.01	9.62	0.680
**N02CC01**	NA	NA	NA	NA	NA
**N02CC03**	1.000	1.00	0.01	78.56	1.000
**N02CC04**	0.500	0.00	0.00	39.00	0.789
**N02CC05**	0.500	0.00	0.00	39.00	0.789
**N02CC06**	NA	NA	NA	NA	NA
**N03AA02**	0.374	3.01	0.24	158.05	0.586
**N03AA03**	1.000	1.00	0.01	78.56	1.000
**N03AB02**	1.000	1.00	0.07	13.83	1.000
**N03AE01**	0.708	1.16	0.51	2.66	0.728
**N03AF01**	1.000	1.00	0.07	13.83	1.000
**N03AF02**	NA	NA	NA	NA	NA
**N03AF04**	0.500	Inf	0.03	Inf	0.789
**N03AG01**	0.507	0.60	0.09	3.09	0.604
**N03AX09**	1.000	1.00	0.07	13.83	1.000
**N03AX11**	0.625	2.00	0.10	118.26	0.680
**N03AX12**	0.305	1.44	0.69	3.11	0.527
**N03AX14**	0.164	2.02	0.70	6.59	0.524
**N03AX15**	0.625	2.00	0.10	118.26	0.680
**N03AX16**	0.717	1.09	0.66	1.82	0.734
**N03AX18**	0.687	0.67	0.06	5.83	0.716
**N03AX22**	0.500	0.00	0.00	39.00	0.789
**N03AX23**	0.500	0.00	0.00	39.00	0.789
**N04AA02**	1.000	1.00	0.07	13.83	1.000
**N04BA02**	0.553	1.20	0.64	2.27	0.620
**N04BB01**	1.000	1.00	0.01	78.56	1.000
**N04BC04**	0.031	0.00	0.00	1.09	0.844
**N04BC05**	0.753	0.80	0.16	3.73	0.766
**N04BC09**	1.000	1.00	0.01	78.56	1.000
**N04BD02**	0.478	1.43	0.49	4.47	0.585
**N04BD03**	0.500	Inf	0.03	Inf	0.789
**N05AA01**	NA	NA	NA	NA	NA
**N05AA02**	0.250	Inf	0.19	Inf	0.803
**N05AD01**	0.790	0.86	0.24	2.99	0.797

**N05AF05**	0.500	0.00	0.00	39.00	0.789
**N05AG02**	NA	NA	NA	NA	NA
**N05AH**	0.125	Inf	0.41	Inf	0.820
**N05AH02**	NA	NA	NA	NA	NA
**N05AH03**	0.726	1.34	0.22	9.15	0.744
**N05AH04**	0.117	1.43	0.89	2.31	0.506
**N05AH06**	0.500	Inf	0.03	Inf	0.789
**N05AL01**	0.342	0.50	0.08	2.34	0.552
**N05AL03**	NA	NA	NA	NA	NA
**N05AL05**	NA	NA	NA	NA	NA
**N05AN01**	1.000	1.00	0.07	13.83	1.000
**N05AX08**	0.210	1.60	0.73	3.64	0.516
**N05AX12**	1.000	1.00	0.01	78.56	1.000
**N05AX13**	0.500	Inf	0.03	Inf	0.789
**N05BA01**	0.478	0.70	0.22	2.04	0.585
**N05BA05**	0.726	0.75	0.11	4.45	0.744
**N05BA06**	0.106	0.77	0.55	1.07	0.503
**N05BA08**	0.922	1.02	0.68	1.53	0.923
**N05BA09**	NA	NA	NA	NA	NA
**N05BA10**	0.500	Inf	0.03	Inf	0.789
**N05BA12**	0.108	0.28	0.03	1.50	0.557
**N05BA14**	NA	NA	NA	NA	NA
**N05BA51**	0.218	4.01	0.40	197.95	0.577
**N05BB01**	0.288	0.40	0.04	2.44	0.554
**N05CD06**	0.105	1.64	0.87	3.16	0.510
**N05CD08**	0.500	0.00	0.00	39.00	0.789
**N05CD09**	0.500	Inf	0.03	Inf	0.789
**N05CD11**	NA	NA	NA	NA	NA
**N05CF01**	NA	NA	NA	NA	NA
**N05CF02**	0.858	1.07	0.49	2.34	0.861
**N05CM02**	0.547	1.50	0.35	7.28	0.622
**N06AA04**	1.000	1.00	0.01	78.56	1.000
**N06AA09**	0.627	1.29	0.42	4.10	0.668
**N06AB03**	0.062	0.00	0.00	1.51	0.833
**N06AB04**	0.844	0.92	0.38	2.21	0.847
**N06AB05**	0.170	1.63	0.77	3.58	0.514
**N06AB06**	0.697	0.90	0.52	1.56	0.719
**N06AB08**	1.000	1.00	0.01	78.56	1.000
**N06AB10**	0.284	0.71	0.36	1.37	0.522
**N06AX05**	0.355	1.21	0.79	1.86	0.534
**N06AX12**	0.625	0.50	0.01	9.62	0.680
**N06AX14**	0.500	0.00	0.00	39.00	0.789
**N06AX16**	0.547	0.66	0.14	2.82	0.622
**N06AX18**	0.500	Inf	0.03	Inf	0.789
**N06AX21**	1.000	1.00	0.48	2.10	1.000
**N06AX23**	0.070	6.04	0.73	278.06	0.595

**N06AX26**	0.288	2.51	0.41	26.45	0.554
**N06BX06**	0.062	0.00	0.00	1.51	0.833
**N06DA02**	0.276	1.51	0.68	3.47	0.523
**N06DA03**	1.000	1.00	0.39	2.56	1.000
**N06DA04**	0.625	0.50	0.01	9.62	0.680
**N06DX01**	0.148	1.89	0.75	5.19	0.519
**N06DX02**	0.250	Inf	0.19	Inf	0.803
**N07AA02**	0.687	1.50	0.17	18.03	0.716
**N07AX01**	0.500	0.00	0.00	39.00	0.789
**N07BA01**	NA	NA	NA	NA	NA
**N07BA03**	0.500	Inf	0.03	Inf	0.789
**N07BC02**	NA	NA	NA	NA	NA
**N07CA01**	0.305	1.44	0.69	3.11	0.527
**N07CA03**	0.250	0.00	0.00	5.32	0.803
**N07XX06**	NA	NA	NA	NA	NA
**P01AB01**	0.500	0.00	0.00	39.00	0.789
**P01AX06**	0.500	0.00	0.00	39.00	0.789
**P01BA01**	0.250	0.00	0.00	5.32	0.803
**P01BA02**	0.422	1.61	0.46	6.27	0.565
**P01BB51**	NA	NA	NA	NA	NA
**R01AC02**	0.500	0.00	0.00	39.00	0.789
**R01AC03**	NA	NA	NA	NA	NA
**R01AD**	0.500	Inf	0.03	Inf	0.789
**R01AD05**	1.000	1.00	0.39	2.56	1.000
**R01AD08**	0.500	Inf	0.03	Inf	0.789
**R01AD09**	0.207	0.57	0.20	1.46	0.521
**R01AD11**	0.500	Inf	0.03	Inf	0.789
**R01AD12**	0.698	0.85	0.36	2.01	0.720
**R01AD58**	NA	NA	NA	NA	NA
**R01AX03**	0.500	Inf	0.03	Inf	0.789
**R02AA52**	NA	NA	NA	NA	NA
**R03AC03**	0.342	0.50	0.08	2.34	0.552
**R03AC12**	0.250	0.00	0.00	5.32	0.803
**R03AC13**	0.500	0.00	0.00	39.00	0.789
**R03AC18**	0.687	0.67	0.06	5.83	0.716
**R03AK**	0.283	0.61	0.22	1.60	0.528
**R03AK06**	0.372	1.39	0.64	3.12	0.543
**R03AK08**	0.062	0.43	0.15	1.12	0.525
**R03AK10**	0.831	0.91	0.34	2.37	0.835
**R03AK11**	1.000	1.00	0.07	13.83	1.000
**R03AL02**	0.288	2.51	0.41	26.45	0.554
**R03AL03**	0.452	2.00	0.29	22.23	0.588
**R03AL04**	0.084	1.76	0.89	3.61	0.513
**R03AL05**	0.452	0.50	0.04	3.49	0.588
**R03AL06**	0.330	1.68	0.55	5.64	0.539
**R03AL08**	0.125	0.00	0.00	2.42	0.820

**R03AL09**	0.062	0.00	0.00	1.51	0.833
**R03BA02**	0.554	0.78	0.32	1.87	0.621
**R03BA05**	0.625	2.00	0.10	118.26	0.680
**R03BA08**	NA	NA	NA	NA	NA
**R03BB04**	0.091	0.64	0.36	1.11	0.508
**R03BB05**	0.164	2.02	0.70	6.59	0.524
**R03BB06**	0.452	0.50	0.04	3.49	0.588
**R03BB07**	0.500	Inf	0.03	Inf	0.789
**R03DA04**	NA	NA	NA	NA	NA
**R03DC03**	0.299	0.55	0.14	1.85	0.536
**R03DX07**	1.000	1.00	0.01	78.56	1.000
**R05CB01**	0.113	0.46	0.14	1.30	0.525
**R05CB03**	0.726	1.34	0.22	9.15	0.744
**R05CB06**	1.000	1.00	0.01	78.56	1.000
**R05CB10**	NA	NA	NA	NA	NA
**R05DA04**	0.179	0.33	0.03	1.86	0.551
**R05DA09**	0.500	0.00	0.00	39.00	0.789
**R05DB21**	1.000	1.00	0.01	78.56	1.000
**R05FA02**	0.500	Inf	0.03	Inf	0.789
**R06AA59**	NA	NA	NA	NA	NA
**R06AB02**	0.478	1.43	0.49	4.47	0.585
**R06AD07**	NA	NA	NA	NA	NA
**R06AE07**	0.091	0.46	0.16	1.21	0.523
**R06AE09**	1.000	1.00	0.01	78.56	1.000
**R06AE57**	NA	NA	NA	NA	NA
**R06AE92**	0.500	0.00	0.00	39.00	0.789
**R06AX22**	0.879	1.05	0.54	2.03	0.880
**R06AX26**	1.000	1.00	0.01	78.56	1.000
**R06AX27**	0.125	Inf	0.41	Inf	0.820
**R06AX28**	1.000	1.00	0.01	78.56	1.000
**R06AX29**	0.838	1.09	0.44	2.75	0.841
**S01AA04**	0.500	Inf	0.03	Inf	0.789
**S01AA12**	1.000	1.00	0.13	7.49	1.000
**S01AA30**	NA	NA	NA	NA	NA
**S01AE01**	0.250	Inf	0.19	Inf	0.803
**S01AE07**	0.500	0.00	0.00	39.00	0.789
**S01BA01**	0.124	5.02	0.56	237.95	0.585
**S01BA02**	NA	NA	NA	NA	NA
**S01BA04**	0.500	Inf	0.03	Inf	0.789
**S01BA07**	0.250	Inf	0.19	Inf	0.803
**S01BA13**	0.500	Inf	0.03	Inf	0.789
**S01BC03**	NA	NA	NA	NA	NA
**S01BC05**	1.000	1.00	0.01	78.56	1.000
**S01CA01**	1.000	1.00	0.19	5.39	1.000
**S01EA05**	0.031	Inf	0.92	Inf	0.844
**S01EC01**	0.500	Inf	0.03	Inf	0.789

**S01EC03**	0.500	Inf	0.03	Inf	0.789
**S01EC04**	0.579	0.71	0.18	2.62	0.639
**S01EC54**	0.452	0.50	0.04	3.49	0.588
**S01ED01**	1.000	1.00	0.19	5.39	1.000
**S01ED05**	NA	NA	NA	NA	NA
**S01ED51**	0.177	0.66	0.34	1.25	0.511
**S01EE01**	0.357	0.70	0.30	1.57	0.539
**S01EE03**	0.813	0.89	0.30	2.61	0.819
**S01EE04**	0.452	0.50	0.04	3.49	0.588
**S01EE05**	NA	NA	NA	NA	NA
**S01FA01**	0.500	Inf	0.03	Inf	0.789
**S01FA06**	0.500	0.00	0.00	39.00	0.789
**S01GX01**	NA	NA	NA	NA	NA
**S01GX02**	0.579	0.71	0.18	2.62	0.639
**S01GX04**	1.000	1.00	0.01	78.56	1.000
**S01GX07**	0.452	2.00	0.29	22.23	0.588
**S01GX08**	1.000	1.00	0.07	13.83	1.000
**S01GX09**	0.500	0.00	0.00	39.00	0.789
**S01GX10**	NA	NA	NA	NA	NA
**S01XA03**	NA	NA	NA	NA	NA
**S01XA20**	0.218	0.25	0.01	2.53	0.577
**S02AA15**	0.500	0.00	0.00	39.00	0.789
**S02BA08**	0.500	Inf	0.03	Inf	0.789
**S02CA05**	1.000	1.00	0.01	78.56	1.000
**S02CA06**	0.250	0.00	0.00	5.32	0.803
**S03CA01**	0.500	Inf	0.03	Inf	0.789
**S03CA04**	0.753	0.80	0.16	3.73	0.766
**V03AE01**	0.386	1.76	0.44	8.22	0.557
**V03AE02**	1.000	1.00	0.19	5.39	1.000
**V03AE03**	0.625	0.50	0.01	9.62	0.680
**V03AE05**	0.374	0.33	0.01	4.15	0.586
**V03AE07**	0.125	0.00	0.00	2.42	0.820
**V03AF03**	0.374	0.33	0.01	4.15	0.586
**V03AF04**	NA	NA	NA	NA	NA

INF. INFINITE; NA. NO ANSWER; OR. ODDS RATIO; P. VALUE. STATISTICAL SIGNIFICANCE

Table S2 Propensity score matching analysis of Subgroup of ATC. Lower and upper limits of the 95% confidence interval for the Odds Ratio.

ATC CODE	DRUG P.VALUE	OR	LOWER LIMIT 95% CI	UPPER LIMIT 95% CI	POWER
**A01AB**	1.000	1.00	0.01	78.56	1.000
**A01AC**	0.250	0.00	0.00	5.32	0.803
**A02AD**	0.500	Inf	0.03	Inf	0.789
**A02AH**	0.500	0.00	0.00	39.00	0.789
**A02BA**	0.803	1.14	0.36	3.73	0.809
**A02BC**	0.257	1.13	0.91	1.41	0.512
**A02BD**	NA	NA	NA	NA	NA
**A02BX**	1.000	1.00	0.01	78.56	1.000
**A03AA**	0.726	1.34	0.22	9.15	0.744
**A03AB**	0.500	0.00	0.00	39.00	0.789
**A03AX**	0.125	Inf	0.41	Inf	0.820
**A03BB**	0.124	0.20	0.00	1.79	0.585
**A03FA**	0.730	0.92	0.57	1.49	0.745
**A04AD**	0.753	0.80	0.16	3.73	0.766
**A05AA**	0.048	0.33	0.08	1.09	0.541
**A06AA**	0.374	0.33	0.01	4.15	0.586
**A06AB**	0.250	0.00	0.00	5.32	0.803
**A06AC**	0.547	0.66	0.14	2.82	0.622
**A06AD**	0.767	0.91	0.48	1.72	0.777
**A06AG**	0.250	0.00	0.00	5.32	0.803
**A06AX**	NA	NA	NA	NA	NA
**A07AA**	0.661	0.82	0.30	2.18	0.692
**A07CA**	<0.001	0.15	0.03	0.53	0.591
**A07DA**	1.000	1.00	0.01	78.56	1.000
**A07EA**	NA	NA	NA	NA	NA
**A07EC**	1.000	1.00	0.19	5.39	1.000
**A07FA**	NA	NA	NA	NA	NA
**A07XA**	1.000	1.00	0.01	78.56	1.000
**A09AA**	1.000	1.00	0.13	7.49	1.000
**A10AB**	0.029	2.04	1.03	4.24	0.517
**A10AC**	0.500	0.00	0.00	39.00	0.789
**A10AD**	0.288	2.51	0.41	26.45	0.554
**A10AE**	0.337	1.23	0.79	1.93	0.530
**A10BA**	0.304	0.82	0.56	1.21	0.522
**A10BB**	0.164	2.02	0.70	6.59	0.524
**A10BD**	0.736	1.08	0.68	1.73	0.750
**A10BF**	NA	NA	NA	NA	NA
**A10BH**	0.397	1.23	0.74	2.06	0.548
**A10BJ**	1.000	1.00	0.13	7.49	1.000
**A10BK**	0.091	2.17	0.83	6.33	0.523
**A10BX**	1.000	1.00	0.42	2.36	1.000
**A11CA**	0.250	Inf	0.19	Inf	0.803

**A11CC**	0.953	1.01	0.79	1.28	0.953
**A11DA**	0.625	2.00	0.10	118.26	0.680
**A11DB**	0.250	0.00	0.00	5.32	0.803
**A11EX**	NA	NA	NA	NA	NA
**A11GA**	0.500	0.00	0.00	39.00	0.789
**A11HA**	1.000	1.00	0.13	7.49	1.000
**A12AA**	0.356	0.63	0.21	1.80	0.543
**A12AX**	0.615	0.92	0.65	1.30	0.658
**A12BA**	0.547	1.50	0.35	7.28	0.622
**A12CC**	0.687	0.67	0.06	5.83	0.716
**A12CX**	0.250	Inf	0.19	Inf	0.803
**A15ZZ**	0.500	Inf	0.03	Inf	0.789
**B01AA**	0.094	1.37	0.93	2.03	0.504
**B01AB**	<0.001	0.12	0.07	0.20	<0.001
**B01AC**	0.070	1.25	0.97	1.62	0.502
**B01AD**	0.500	0.00	0.00	39.00	0.789
**B01AE**	1.000	1.00	0.27	3.76	1.000
**B01AF**	0.835	1.04	0.68	1.61	0.838
**B01AX**	NA	NA	NA	NA	NA
**B02AA**	0.500	Inf	0.03	Inf	0.789
**B03AA**	0.249	1.28	0.83	1.98	0.514
**B03AB**	0.838	1.09	0.44	2.75	0.841
**B03BA**	0.210	1.34	0.83	2.17	0.510
**B03BB**	0.001	2.32	1.36	4.08	0.520
**B05BB**	0.250	0.00	0.00	5.32	0.803
**C01AA**	0.011	3.81	1.20	15.84	0.553
**C01BC**	1.000	1.00	0.13	7.49	1.000
**C01BD**	1.000	1.00	0.19	5.39	1.000
**C01CA**	0.500	Inf	0.03	Inf	0.789
**C01DA**	0.815	0.95	0.58	1.54	0.820
**C01EB**	0.538	1.31	0.52	3.35	0.612
**C02AC**	0.500	Inf	0.03	Inf	0.789
**C02CA**	0.177	1.52	0.80	2.96	0.511
**C02DB**	0.500	0.00	0.00	39.00	0.789
**C02DC**	NA	NA	NA	NA	NA
**C03AA**	0.698	1.17	0.50	2.79	0.720
**C03BA**	0.698	1.17	0.50	2.79	0.720
**C03CA**	0.168	1.21	0.91	1.60	0.504
**C03DA**	0.459	1.20	0.72	2.03	0.571
**C03EA**	0.646	1.25	0.44	3.68	0.681
**C04AD**	0.208	2.01	0.62	7.55	0.528
**C05AA**	NA	NA	NA	NA	NA
**C05AE**	0.125	0.00	0.00	2.42	0.820
**C05AX**	0.500	0.00	0.00	39.00	0.789
**C05BX**	0.500	0.00	0.00	39.00	0.789
**C05CA**	0.125	0.00	0.00	2.42	0.820

**C07AA**	0.605	0.75	0.21	2.47	0.654
**C07AB**	0.773	1.04	0.78	1.40	0.782
**C07AG**	0.058	1.70	0.95	3.11	0.510
**C07BB**	NA	NA	NA	NA	NA
**C07CB**	0.250	Inf	0.19	Inf	0.803
**C08CA**	0.812	0.96	0.70	1.33	0.817
**C08DA**	0.687	0.67	0.06	5.83	0.716
**C08DB**	0.386	1.76	0.44	8.22	0.557
**C09AA**	0.209	0.84	0.63	1.11	0.507
**C09BA**	0.032	1.64	1.02	2.68	0.508
**C09BB**	1.000	1.00	0.23	4.37	1.000
**C09CA**	0.656	1.08	0.75	1.56	0.687
**C09DA**	0.536	0.88	0.57	1.35	0.608
**C09DB**	0.661	0.82	0.30	2.18	0.692
**C09DX**	0.399	1.34	0.65	2.85	0.550
**C09XA**	0.625	2.00	0.10	118.26	0.680
**C10AA**	0.013	1.32	1.05	1.65	0.502
**C10AB**	0.323	1.51	0.63	3.79	0.533
**C10AC**	1.000	1.00	0.13	7.49	1.000
**C10AX**	0.613	1.19	0.58	2.50	0.657
**C10BA**	0.050	0.54	0.27	1.04	0.513
**C10BX**	0.031	Inf	0.92	Inf	0.844
**D01AC**	0.863	0.94	0.44	2.00	0.865
**D01AE**	0.757	0.91	0.46	1.76	0.769
**D01BA**	NA	NA	NA	NA	NA
**D03BA**	0.374	3.01	0.24	158.05	0.586
**D05AX**	0.034	3.71	0.97	20.78	0.554
**D05BB**	0.250	Inf	0.19	Inf	0.803
**D05BX**	NA	NA	NA	NA	NA
**D06AX**	0.117	0.40	0.09	1.38	0.534
**D06BA**	0.062	Inf	0.66	Inf	0.833
**D06BB**	NA	NA	NA	NA	NA
**D06BX**	1.000	1.00	0.01	78.56	1.000
**D07AA**	0.625	0.50	0.01	9.62	0.680
**D07AB**	1.000	1.00	0.01	78.56	1.000
**D07AC**	0.258	0.77	0.48	1.24	0.515
**D07AD**	0.554	0.78	0.32	1.87	0.621
**D07CA**	NA	NA	NA	NA	NA
**D07CB**	NA	NA	NA	NA	NA
**D07CC**	0.500	Inf	0.03	Inf	0.789
**D07XC**	0.625	0.50	0.01	9.62	0.680
**D09AA**	0.500	Inf	0.03	Inf	0.789
**D10AF**	1.000	1.00	0.01	78.56	1.000
**D10BA**	NA	NA	NA	NA	NA
**D11AH**	0.627	0.78	0.24	2.36	0.668
**D11AX**	1.000	1.00	0.19	5.39	1.000

**G01AC**	NA	NA	NA	NA	NA
**G01AF**	0.124	0.20	0.00	1.79	0.585
**G02BB**	NA	NA	NA	NA	NA
**G02CB**	NA	NA	NA	NA	NA
**G02CC**	NA	NA	NA	NA	NA
**G03AA**	NA	NA	NA	NA	NA
**G03AB**	NA	NA	NA	NA	NA
**G03AC**	NA	NA	NA	NA	NA
**G03AD**	NA	NA	NA	NA	NA
**G03BA**	0.500	Inf	0.03	Inf	0.789
**G03CA**	NA	NA	NA	NA	NA
**G03CX**	NA	NA	NA	NA	NA
**G03DA**	NA	NA	NA	NA	NA
**G03DC**	NA	NA	NA	NA	NA
**G03FB**	NA	NA	NA	NA	NA
**G03HA**	0.500	Inf	0.03	Inf	0.789
**G03HB**	NA	NA	NA	NA	NA
**G03XC**	NA	NA	NA	NA	NA
**G04BC**	0.500	Inf	0.03	Inf	0.789
**G04BD**	0.245	1.42	0.76	2.68	0.516
**G04BE**	0.374	0.33	0.01	4.15	0.586
**G04CA**	0.136	1.24	0.93	1.66	0.503
**G04CB**	0.774	1.20	0.30	5.00	0.783
**G04CX**	0.131	2.02	0.76	5.95	0.522
**H01BA**	NA	NA	NA	NA	NA
**H01CB**	0.500	0.00	0.00	39.00	0.789
**H02AA**	1.000	1.00	0.01	78.56	1.000
**H02AB**	0.429	1.17	0.78	1.77	0.558
**H03AA**	0.227	0.81	0.57	1.16	0.510
**H03BB**	0.726	1.34	0.22	9.15	0.744
**H03CA**	NA	NA	NA	NA	NA
**H04AA**	0.726	1.34	0.22	9.15	0.744
**H05AA**	0.625	2.00	0.10	118.26	0.680
**H05BA**	NA	NA	NA	NA	NA
**H05BX**	0.507	1.67	0.32	10.80	0.604
**J01AA**	0.250	0.00	0.00	5.32	0.803
**J01CA**	0.858	0.94	0.43	2.04	0.861
**J01CE**	NA	NA	NA	NA	NA
**J01CF**	0.218	0.25	0.01	2.53	0.577
**J01CR**	0.074	0.58	0.29	1.10	0.512
**J01DB**	0.500	0.00	0.00	39.00	0.789
**J01DC**	0.011	0.26	0.06	0.83	0.553
**J01DD**	0.844	0.92	0.38	2.21	0.847
**J01EE**	0.605	1.34	0.40	4.70	0.654
**J01FA**	0.001	0.44	0.25	0.75	0.519
**J01FF**	1.000	1.00	0.07	13.83	1.000

**J01GB**	0.500	0.00	0.00	39.00	0.789
**J01MA**	0.039	0.62	0.37	1.00	0.508
**J01XE**	0.726	1.34	0.22	9.15	0.744
**J01XX**	0.646	0.87	0.45	1.67	0.680
**J02AC**	0.288	0.40	0.04	2.44	0.554
**J04AB**	NA	NA	NA	NA	NA
**J04AC**	NA	NA	NA	NA	NA
**J05AB**	0.753	0.80	0.16	3.73	0.766
**J05AH**	0.500	0.00	0.00	39.00	0.789
**L01BA**	1.000	1.00	0.01	78.56	1.000
**L01BC**	0.625	0.50	0.01	9.62	0.680
**L01XX**	0.625	0.50	0.01	9.62	0.680
**L02AB**	1.000	1.00	0.01	78.56	1.000
**L02AE**	0.131	0.50	0.17	1.32	0.522
**L02BA**	1.000	1.00	0.01	78.56	1.000
**L02BB**	1.000	1.00	0.07	13.83	1.000
**L02BG**	1.000	1.00	0.19	5.39	1.000
**L04AA**	0.386	1.76	0.44	8.22	0.557
**L04AD**	0.039	7.05	0.90	318.27	0.604
**L04AX**	0.148	1.89	0.75	5.19	0.519
**M01AB**	1.000	1.00	0.13	7.49	1.000
**M01AC**	0.250	0.00	0.00	5.32	0.803
**M01AE**	0.096	0.67	0.40	1.10	0.507
**M01AG**	NA	NA	NA	NA	NA
**M01AH**	0.342	2.01	0.43	12.46	0.552
**M01AX**	0.030	0.30	0.07	0.99	0.545
**M01CX**	0.250	0.00	0.00	5.32	0.803
**M02AA**	0.500	0.00	0.00	39.00	0.789
**M03BA**	0.500	Inf	0.03	Inf	0.789
**M03BX**	0.145	0.37	0.06	1.56	0.540
**M04AA**	0.018	1.46	1.06	2.03	0.504
**M04AC**	0.208	0.50	0.13	1.60	0.528
**M05BA**	1.000	1.00	0.44	2.28	1.000
**M05BB**	0.625	2.00	0.10	118.26	0.680
**M05BX**	0.866	1.06	0.51	2.21	0.868
**N01BA**	NA	NA	NA	NA	NA
**N01BB**	1.000	1.00	0.19	5.39	1.000
**N01BX**	0.452	2.00	0.29	22.23	0.588
**N02AA**	0.113	2.19	0.77	7.06	0.525
**N02AB**	0.050	2.28	0.94	6.11	0.524
**N02AE**	0.374	0.33	0.01	4.15	0.586
**N02AJ**	0.056	0.70	0.48	1.02	0.504
**N02AX**	0.886	1.04	0.57	1.93	0.887
**N02BA**	0.500	Inf	0.03	Inf	0.789
**N02BB**	0.806	1.03	0.80	1.32	0.812
**N02BE**	0.448	1.09	0.87	1.35	0.565

**N02BG**	0.500	0.00	0.00	39.00	0.789
**N02CA**	0.625	0.50	0.01	9.62	0.680
**N02CC**	0.374	0.33	0.01	4.15	0.586
**N03AA**	0.452	2.00	0.29	22.23	0.588
**N03AB**	1.000	1.00	0.07	13.83	1.000
**N03AE**	0.708	1.16	0.51	2.66	0.728
**N03AF**	0.687	1.50	0.17	18.03	0.716
**N03AG**	0.507	0.60	0.09	3.09	0.604
**N03AX**	0.144	1.31	0.90	1.91	0.505
**N04AA**	1.000	1.00	0.07	13.83	1.000
**N04BA**	0.553	1.20	0.64	2.27	0.620
**N04BB**	1.000	1.00	0.01	78.56	1.000
**N04BC**	0.141	0.45	0.12	1.42	0.528
**N04BD**	0.356	1.58	0.56	4.84	0.543
**N05AA**	0.250	Inf	0.19	Inf	0.803
**N05AD**	0.790	0.86	0.24	2.99	0.797
**N05AF**	0.500	0.00	0.00	39.00	0.789
**N05AG**	NA	NA	NA	NA	NA
**N05AH**	0.105	1.43	0.91	2.26	0.506
**N05AL**	0.342	0.50	0.08	2.34	0.552
**N05AN**	1.000	1.00	0.07	13.83	1.000
**N05AX**	0.170	1.63	0.77	3.58	0.514
**N05BA**	0.195	0.85	0.65	1.10	0.506
**N05BB**	0.288	0.40	0.04	2.44	0.554
**N05CD**	0.080	1.70	0.91	3.27	0.511
**N05CF**	0.858	1.07	0.49	2.34	0.861
**N05CM**	0.547	1.50	0.35	7.28	0.622
**N06AA**	0.646	1.25	0.44	3.68	0.681
**N06AB**	0.567	0.91	0.65	1.27	0.626
**N06AX**	0.015	1.44	1.06	1.95	0.504
**N06BX**	0.062	0.00	0.00	1.51	0.833
**N06DA**	0.496	1.21	0.68	2.17	0.588
**N06DX**	0.073	2.15	0.87	5.80	0.522
**N07AA**	0.687	1.50	0.17	18.03	0.716
**N07AX**	0.500	0.00	0.00	39.00	0.789
**N07BA**	0.500	Inf	0.03	Inf	0.789
**N07BC**	NA	NA	NA	NA	NA
**N07CA**	0.506	1.26	0.61	2.62	0.594
**N07XX**	NA	NA	NA	NA	NA
**P01AB**	0.500	0.00	0.00	39.00	0.789
**P01AX**	0.500	0.00	0.00	39.00	0.789
**P01BA**	0.605	1.34	0.40	4.70	0.654
**P01BB**	NA	NA	NA	NA	NA
**R01AC**	0.500	0.00	0.00	39.00	0.789
**R01AD**	0.546	0.86	0.52	1.43	0.614
**R01AX**	0.500	Inf	0.03	Inf	0.789

**R02AA**	NA	NA	NA	NA	NA
**R03AC**	0.010	0.57	0.36	0.90	0.510
**R03AK**	0.023	0.68	0.48	0.96	0.505
**R03AL**	0.247	1.31	0.81	2.15	0.514
**R03BA**	0.708	0.86	0.38	1.96	0.728
**R03BB**	0.023	0.67	0.46	0.96	0.505
**R03DA**	0.015	0.00	0.00	0.85	0.853
**R03DC**	0.299	0.55	0.14	1.85	0.536
**R03DX**	1.000	1.00	0.01	78.56	1.000
**R05CB**	0.181	0.58	0.24	1.36	0.517
**R05DA**	0.108	0.28	0.03	1.50	0.557
**R05DB**	1.000	1.00	0.01	78.56	1.000
**R05FA**	0.500	Inf	0.03	Inf	0.789
**R06AA**	NA	NA	NA	NA	NA
**R06AB**	0.478	1.43	0.49	4.47	0.585
**R06AD**	NA	NA	NA	NA	NA
**R06AE**	0.073	0.46	0.17	1.15	0.522
**R06AX**	0.655	0.90	0.57	1.44	0.686
**S01AA**	0.726	1.34	0.22	9.15	0.744
**S01AE**	0.625	2.00	0.10	118.26	0.680
**S01BA**	0.039	7.05	0.90	318.27	0.604
**S01BC**	1.000	1.00	0.01	78.56	1.000
**S01CA**	1.000	1.00	0.19	5.39	1.000
**S01EA**	0.031	Inf	0.92	Inf	0.844
**S01EC**	0.823	0.90	0.32	2.48	0.827
**S01ED**	0.263	0.72	0.39	1.31	0.518
**S01EE**	0.263	0.72	0.39	1.31	0.518
**S01FA**	1.000	1.00	0.01	78.56	1.000
**S01GX**	0.844	0.92	0.38	2.21	0.847
**S01XA**	0.218	0.25	0.01	2.53	0.577
**S02AA**	0.500	0.00	0.00	39.00	0.789
**S02BA**	0.500	Inf	0.03	Inf	0.789
**S02CA**	0.625	0.50	0.01	9.62	0.680
**S03CA**	1.000	1.00	0.23	4.37	1.000
**V03AE**	1.000	1.00	0.39	2.56	1.000
**V03AF**	0.374	0.33	0.01	4.15	0.586
**INF. INFINITE; NA. NO ANSWER; OR. ODDS RATIO; P. VALUE. STATISTICAL SIGNIFICANCE**					

Table S3 Propensity score matching analysis of the main therapeutic group. Lower and upper limits of the 95% confidence interval for the Odds Ratio.

ATC CODE	DRUG P.VALUE	OR	LOWER LIMIT 95% CI	UPPER LIMIT 95% CI	POWER
**A01**	0.374	0.33	0.01	4.15	0.586
**A02**	0.234	1.14	0.91	1.41	0.509
**A03**	0.594	0.89	0.57	1.39	0.644
**A04**	0.753	0.80	0.16	3.73	0.766
**A05**	0.048	0.33	0.08	1.09	0.541
**A06**	0.302	0.76	0.44	1.31	0.523
**A07**	0.033	0.53	0.28	0.99	0.513
**A09**	1.000	1.00	0.13	7.49	1.000
**A10**	0.255	1.16	0.89	1.49	0.512
**A11**	1.000	1.00	0.79	1.27	1.000
**A12**	0.531	0.91	0.66	1.25	0.606
**A15**	0.500	Inf	0.03	Inf	0.789
**B01**	0.160	0.86	0.69	1.07	0.503
**B02**	0.500	Inf	0.03	Inf	0.789
**B03**	<0.001	1.68	1.25	2.26	0.507
**B05**	0.250	0.00	0.00	5.32	0.803
**C01**	0.169	1.31	0.88	1.98	0.506
**C02**	0.137	1.58	0.83	3.06	0.510
**C03**	0.264	1.15	0.89	1.48	0.514
**C04**	0.208	2.01	0.62	7.55	0.528
**C05**	0.004	0.00	0.00	0.58	0.868
**C07**	0.242	1.17	0.89	1.52	0.511
**C08**	0.939	0.99	0.72	1.35	0.939
**C09**	0.828	1.02	0.82	1.27	0.832
**C10**	0.082	1.21	0.97	1.50	0.501
**D01**	0.810	0.94	0.57	1.56	0.816
**D03**	0.374	3.01	0.24	158.05	0.586
**D05**	0.034	3.71	0.97	20.78	0.554
**D06**	0.661	0.82	0.30	2.18	0.692
**D07**	0.270	0.80	0.53	1.21	0.516
**D09**	0.500	Inf	0.03	Inf	0.789
**D10**	1.000	1.00	0.01	78.56	1.000
**D11**	0.838	0.92	0.36	2.28	0.841
**G01**	0.124	0.20	0.00	1.79	0.585
**G02**	NA	NA	NA	NA	NA
**G03**	0.250	Inf	0.19	Inf	0.803
**G04**	0.100	1.24	0.95	1.62	0.502
**H01**	0.500	0.00	0.00	39.00	0.789
**H02**	0.375	1.19	0.79	1.80	0.540
**H03**	0.272	0.83	0.58	1.17	0.516
**H04**	0.726	1.34	0.22	9.15	0.744

**H05**	0.386	1.76	0.44	8.22	0.557
**J01**	<0.001	0.53	0.40	0.70	0.510
**J02**	0.288	0.40	0.04	2.44	0.554
**J04**	NA	NA	NA	NA	NA
**J05**	0.547	0.66	0.14	2.82	0.622
**L01**	0.507	0.60	0.09	3.09	0.604
**L02**	0.224	0.64	0.29	1.37	0.517
**L04**	0.095	1.80	0.86	3.89	0.514
**M01**	0.050	0.66	0.43	1.02	0.506
**M02**	0.500	0.00	0.00	39.00	0.789
**M03**	0.265	0.50	0.11	1.87	0.536
**M04**	0.015	1.47	1.06	2.03	0.504
**M05**	0.800	1.07	0.63	1.83	0.806
**N01**	0.605	1.34	0.40	4.70	0.654
**N02**	0.824	1.03	0.82	1.28	0.828
**N03**	0.162	1.26	0.90	1.77	0.505
**N04**	0.697	1.11	0.64	1.92	0.719
**N05**	0.955	0.99	0.79	1.25	0.955
**N06**	0.120	1.20	0.95	1.54	0.502
**N07**	0.444	1.27	0.66	2.48	0.566
**P01**	1.000	1.00	0.33	3.08	1.000
**R01**	0.551	0.87	0.52	1.43	0.617
**R02**	NA	NA	NA	NA	NA
**R03**	0.009	0.71	0.55	0.93	0.503
**R05**	0.103	0.57	0.27	1.17	0.513
**R06**	0.390	0.85	0.57	1.26	0.544
**S01**	0.144	0.78	0.54	1.10	0.504
**S02**	0.687	0.67	0.06	5.83	0.716
**S03**	1.000	1.00	0.23	4.37	1.000
**V03**	0.698	0.85	0.36	2.01	0.720
**INF. INFINITE; NA. NO ANSWER; OR. ODDS RATIO; P. VALUE. STATISTICAL SIGNIFICANCE**					
